# Nek2 activation of Kif24 ensures cilium disassembly during the cell cycle

**DOI:** 10.1038/ncomms9087

**Published:** 2015-08-20

**Authors:** Sehyun Kim, Kwanwoo Lee, Jung-Hwan Choi, Niels Ringstad, Brian David Dynlacht

**Affiliations:** 1 Department of Pathology, New York University Cancer Institute, New York University School of Medicine, New York, New York 10016, USA; 2 Skirball Institute of Biomolecular Medicine, Molecular Neurobiology Program, New York University School of Medicine, New York, New York 10016, USA; 3 Department of Cell Biology, New York University School of Medicine, New York, New York 10016, USA

## Abstract

Many proteins are known to promote ciliogenesis, but mechanisms that promote primary cilia disassembly before mitosis are largely unknown. Here we identify a mechanism that favours cilium disassembly and maintains the disassembled state. We show that co-localization of the S/G2 phase kinase, Nek2 and Kif24 triggers Kif24 phosphorylation, inhibiting cilia formation. We show that Kif24, a microtubule depolymerizing kinesin, is phosphorylated by Nek2, which stimulates its activity and prevents the outgrowth of cilia in proliferating cells, independent of Aurora A and HDAC6. Our data also suggest that cilium assembly and disassembly are in dynamic equilibrium, but Nek2 and Kif24 can shift the balance toward disassembly. Further, Nek2 and Kif24 are overexpressed in breast cancer cells, and ablation of these proteins restores ciliation in these cells, thereby reducing proliferation. Thus, Kif24 is a physiological substrate of Nek2, which regulates cilia disassembly through a concerted mechanism involving Kif24-mediated microtubule depolymerization.

## Abstract (web summary)

Most differentiated mammalian cells assemble a primary cilium, which serves as a cellular ‘antenna’ for sensing and responding to the extracellular environment. Here the authors show that Nek2-mediated phosphorylation of Kif24 further promotes the loss of primary cilia, triggered by Aurora A and HDAC6 on cell cycle re-entry.

## Graphical abstract

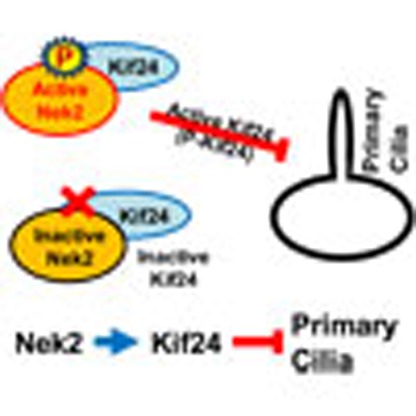

Most quiescent and differentiated mammalian cells assemble a primary cilium, a microtubule-based projection from the cell surface. The cilium serves as a cellular ‘antenna’ for sensing and responding to the extracellular environment. Primary cilia are formed in quiescent cells, and they are resorbed as cells receive mitogenic signals^1^,^2^,^3^,^4^. Ciliary disassembly provoked by growth factor stimulation involves the activation of histone deacetylase 6 (HDAC6) at the axoneme through the concerted action of human enhancer of filamentation 1 and the Aurora A kinase^1^,^3^. Deacetylation of axonemal microtubules results in destabilization of axonemal microtubules, facilitating ciliary retraction, and inhibition of Aurora A or HDAC6 blocks serum-induced ciliary resorption^3^.

Defects in the primary cilium have been shown to cause a spectrum of diseases, including developmental defects, obesity and polycystic kidney disease, which are collectively recognized as ciliopathies^1^,^5^. Defects in primary cilium assembly are also implicated in tumorigenesis, since loss of cilia is commonly associated with multiple types of cancer, including breast, pancreatic and prostatic tumours^6^,^7^,^8^,^9^,^10^. Recently, it was shown that mammary epithelial cells lose primary cilia as they undergo oncogenic transformation^8^,^10^. Breast cancers are classified into several subtypes based on gene expression profiles^11^. The basal subtypes, which include triple-negative breast cancers, have been shown to ciliate, albeit with very low frequency^8^,^10^. These cells are thought to originate from the myoepithelial layer of the mammary gland, which is highly ciliated in both mouse and human tissue^8^,^10^,^12^. Therefore, it is thought that the basal B subtype of breast cancer cells retains the intrinsic machinery to form primary cilia^10^. Nevertheless, whether ciliary dysfunction is a cause or a consequence of cellular transformation is not known.

Recently, several studies have shown that Nek2, an S/G2 phase kinase, is overexpressed in diverse forms of cancer, where it functions as an oncogene^13^,^14^,^15^. Nek2 overexpression leads to increased proliferation and drug resistance of cancer cells, whereas depletion of Nek2 reverts these effects, although the mechanistic role of Nek2 in cancer development is largely unknown^13^,^14^,^15^. Nek2 proteins are encoded by at least two major splice variants, Nek2A and Nek2B (collectively referred to here as Nek2), which differ at their carboxy-termini but exhibit overlapping or identical substrate usage^16^,^17^. Nek2 expression, which is not detectable in G1, increases in S phase and peaks in G2 phase, when it plays an established role in regulating centrosome separation^17^,^18^,^19^. Nek2 has also been implicated in suppression of primary cilium formation, although mechanistic details supporting this role are lacking^20^.

Here we show that Kif24 is a key physiological substrate of Nek2 and that Nek2 negatively regulates ciliogenesis by enhancing Kif24 activity. Previously, Kif24 was shown to act as a centriole-bound, microtubule-depolymerizing kinesin that suppresses primary cilia formation^21^, but its regulation was not well understood. We find that Nek2 stably interacts with, and phosphorylates Kif24, stimulating its microtubule-depolymerizing activity. We also provide evidence that Nek2-dependent phosphorylation induces a conformational change in Kif24 that promotes its activity. Importantly, we show that Nek2–Kif24 plays a role temporally distinct from the well-established Aurora A-HDAC6 ciliary disassembly pathway by blocking the growth of new cilia and nucleation of this structure from basal bodies that have resorbed their cilia. Finally, we found that depletion of either Nek2 or Kif24 in breast cancer cell lines restored ciliation and reduced proliferation of these cells. Our results suggest the potential to target these enzymes in tumour cells.

## Results

### Nek2 binds and phosphorylates Kif24

**Figure 1:**
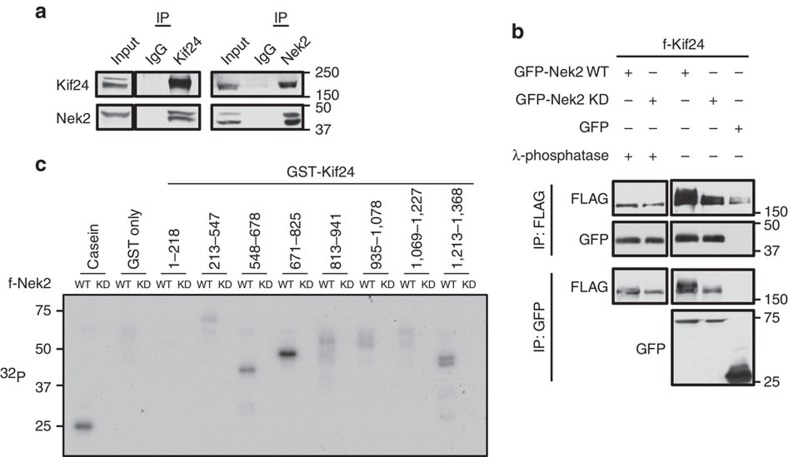
Nek2 stably interacts with, and phosphorylates, Kif24. (**a**) Lysates of HEK293 cells were immunoprecipitated and immuno-blotted with the indicated antibodies to demonstrate reciprocal interactions between Nek2 and Kif24. IgG indicates negative control derived from rabbit (Left panel) or mouse (Right panel). Input and immunoprecipitated samples were electrophoresed on the same gels. (**b**) The indicated Flag- and GFP-tagged proteins were expressed in HEK293 cells and immunoprecipitated, after which they were treated with λ phosphatase or left untreated, and immuno-blotted with anti-Flag and GFP antibodies as indicated. (**c**) *In vitro* kinase assays were performed with the indicated fragments of Kif24 as GST fusions and purified Flag-tagged WT or KD Nek2. Uncropped western blots are shown in Supplementary Fig. 13.

To understand the molecular mechanisms involved in the regulation of primary cilium assembly and disassembly, we employed an unbiased proteomic screen and published data to identify interacting partners of Kif24 (ref. 22). This strategy allowed us to identify Nek2, a serine/threonine kinase previously implicated in centrosome separation and ciliary disassembly^17^,^19^,^20^. We confirmed the interaction between these proteins by co-immunoprecipitation of green fluorescent protein (GFP)-tagged Nek2 (GFP–Nek2) and flag-tagged Kif24 (f–Kif24) from transiently transfected cells and by reciprocal co-immunoprecipitation of endogenous Nek2 and Kif24 (Fig. 1a,b). We generated an array of Kif24 deletion mutants and examined interactions with Nek2, which allowed us to demonstrate that Kif24 interacts with Nek2 through two regions encompassing residues 509–547 and 924–999 (Supplementary Fig. 1).

Next we determined whether Kif24 is a substrate for Nek2. A slower migrating Kif24 species was observed when co-expressed with an active, wild-type (WT) form of Nek2 (Nek2 WT), but not with a catalytically inactive (KD) mutant (Nek2-KD; Fig. 1b). Moreover, treatment of Kif24 with λ phosphatase after co-immunoprecipitation resulted in loss of this slower migrating species (Fig. 1b), confirming that the reduced mobility was due to phosphorylation (Fig. 1b). Further, we performed *in vitro* kinase assays using glutathione *S*-transferase (GST)-tagged Kif24 fragments produced in bacteria and flag-tagged WT or Nek2-KD purified from HEK293 cells, which confirmed that Kif24 was directly phosphorylated by Nek2 (Fig. 1c). Fragments containing residues 548–825 were most robustly phosphorylated, with an efficiency comparable to casein, a canonical substrate of Nek2 (Fig. 1c and Supplementary Figs 1D and 2). However, fragments encompassing regions spanning from the N-terminus to the catalytic motor domain (1–447) were not overtly phosphorylated, suggesting that phospho-dependent control of Kif24 is restricted to regions carboxy-terminal to the kinesin domain (548–825) that are flanked by dedicated Nek2-binding surfaces (Fig. 1c and Supplementary Fig. 1D).

### Cell cycle-dependent co-localization of Nek2 and Kif24

**Figure 2:**
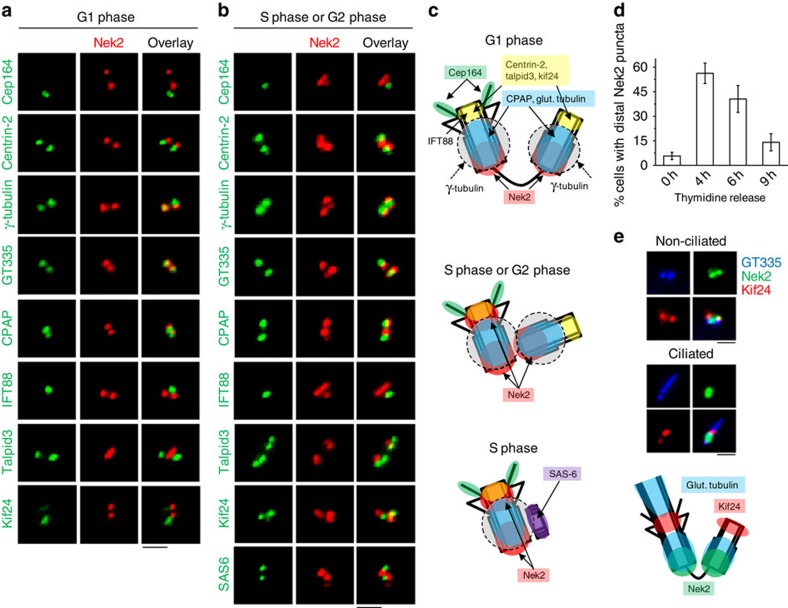
Nek2 and Kif24 co-localize in a cell cycle-dependent manner. (**a**,**b**) Nek2 and Kif24 were visualized with pericentriolar (γ-tubulin), distal (IFT88, Cep164, centrin-2 and Talpid3), and proximal (GT335, SAS6 and CPAP) centriolar markers during G1, S and G2 phases of the cell cycle. Cep164 and IFT88 localize to mother centrioles. Scale bar: 1.5μm. Results are schematized in (**c**). (**d**) Quantitation of cells with Nek2 and Kif24 overlap in cells treated with (0 h), and released from, a double-thymidine block for the indicated number of hours. (**e**) Visualization of Nek2 and Kif24 in ciliated (schematized below) and non-ciliated cells stained with GT335, Nek2 and Kif24 antibodies at 18 h after serum re-stimulation. Representative images are shown. Data were obtained from three biologically independent experiments. Scale bar: 1.5μm. Error bars show s.d.

Nek2 has been extensively studied in the context of centrosome separation during mitosis^19^,^23^. In these studies, Nek2 was reported to localize to the proximal ends of both mother and daughter centrioles^19^. Nek2 has also been shown to localize in a cell cycle-dependent manner to the sub-distal and/or distal ends of the centriole and basal body, where it has been implicated in primary cilium disassembly^20^. We employed a panel of centriolar markers to more precisely define the localization pattern of Nek2 within the centriole throughout the cell cycle in RPE1 cells. We found that Nek2 had two distinct patterns of localization. First, during G1 phase of the cell cycle, Nek2 could be distinguished as two foci separable from centriole distal markers, including Cep164, centrin-2, IFT88, Talpid3 and Kif24, and it overlapped with glutamylated tubulin (GT335) and Centrosomal P4.1-associated protein (CPAP), which marks the proximal region of centrioles, and γ-tubulin at the pericentriolar matrix (Fig. 2a,c). However, during S/G2 phases, we observed an additional (third) dot of Nek2 apart from the two foci localized near the proximal ends of centrioles (marked by GT335, CPAP and γ-tubulin; Fig. 2b,c). We found that this third dot preferentially localized to the mother centriole, as determined by its overlap with Cep164 and IFT88 (Fig. 2b,c). The localization pattern, proximal to Cep164 but overlapping with IFT88, centrin-2, Talpid3 and Kif24, further indicated that Nek2 is concentrated near the distal tip of mother centrioles. In addition, visualization with antibodies against SAS6, which demarcates pro-centrioles during S phase^24^, showed that Nek2 was absent from nascent centrioles, whereas it was present at mature centrioles (Fig. 2b,c). Cell cycle synchronization studies revealed that the additional Nek2 dot co-localized with Kif24 and was prevalent during the S/G2 phases of the cell cycle, but it diminished on mitotic entry (Fig. 2d). Western blot analysis of synchronized cells showed a step-wise reduction in electrophoretic mobility of Kif24 from S to G2 phase, coinciding with the time when Nek2 co-localized with Kif24 (Supplementary Fig. 3A). We conclude that Nek2 co-localizes with Kif24 principally during S/G2 phases and that Kif24 is likely to be a physiological substrate of this kinase.

### Nek2-mediated phosphorylation of Kif24 blocks ciliation

Our previous studies demonstrated that Kif24 regulates ciliogenesis via microtubule depolymerization^21^. Therefore, we sought to test functional links between Nek2 phosphorylation and cilium assembly. Primary cilium assembly and disassembly can be recapitulated *in vitro* using retinal pigment epithelial (RPE1) cells, wherein 48 h of serum starvation leads to robust ciliation, and re-stimulation of cells with serum-containing media (termed serum re-stimulation) leads to cilium disassembly (Supplementary Fig. 3B)^3^. We found that Nek2 expression steadily declined following serum deprivation and gradually re-appeared after 18–24 h of serum stimulation (Supplementary Fig. 3C). Kif24 expression mirrored that of Nek2 (Supplementary Fig. 3C)^21^. Most of the cells lacking cilia at 18 and 24 h post-serum re-stimulation (S and G2/M phases, respectively) showed an overlap between Nek2 and Kif24 at the distal end of the mother centriole (Fig. 2e, and Supplementary Fig. 3C,D). We also found a fraction of ciliated cells that expressed both Nek2 and Kif24 during these time points, but localization of both proteins was largely non-overlapping in ∼76–86% of these re-stimulated, ciliated cells (Fig. 2e, ciliated panel). This supports our observation that Nek2 and Kif24 physically interact and co-localize during a portion of the cell cycle (S and G2/M) in which cells generally lack cilia, but not at other times (G1), prompting us to test a functional connection between these proteins.

**Figure 3:**
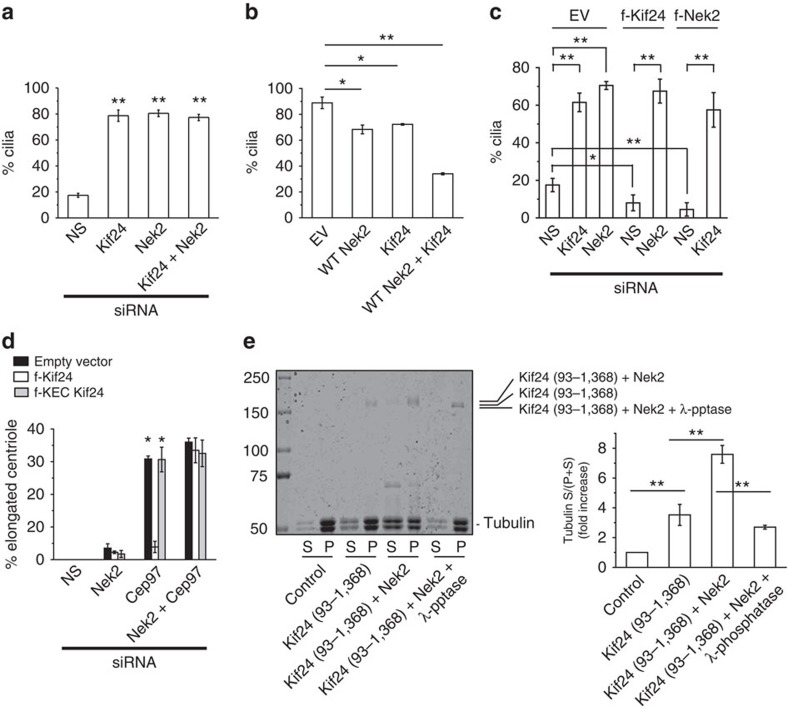
Nek2 is required to stimulate Kif24 microtubule depolymerization. (**a**) Ciliation was examined after depletion of Kif24 and/or Nek2 in growing RPE1 cells. (**b**) Frequency of ciliation was measured as in (**a**) after expression of the indicated proteins. (**c**) RPE1 cells were treated with the indicated siRNAs and transfected to overexpress Flag-tagged Kif24 (f–Kif24) or f–Nek2 as shown. (**d**) U2OS cells were depleted of Cep97 and/or Nek2 with ectopic expression of f–Kif24, or f–KEC Kif24, as indicated, and the per cent of cells with elongated centrioles was measured. (**e**) (Left) *In vitro* microtubule-depolymerization assays were performed with polymerized tubulins and the indicated purified recombinant proteins, which were either treated with λ phosphatase or left untreated. The reaction products were separated into supernatant (s) and pellet (p) fractions to measure release of free tubulins. The migration of phosphorylated and unphosphorylated Kif24 and tubulins is shown. (Right) Quantitation of data shown at left. Tubulin alone was used as control. Fold increase of Tubulin S/(P+S) per sample is normalized to the Tubulin alone control. Data were obtained from three biologically independent experiments. Error bars show s.e.m. *P<0.05, **P<0.001. Statistical significance was tested by comparisons with NS siRNA (**a**) or f–Kif24 (**d**), or as indicated by bars in **b**, **c** and **e**. Uncropped western blots are shown in Supplementary Fig. 13. EV, empty vector control.

**Figure 4:**
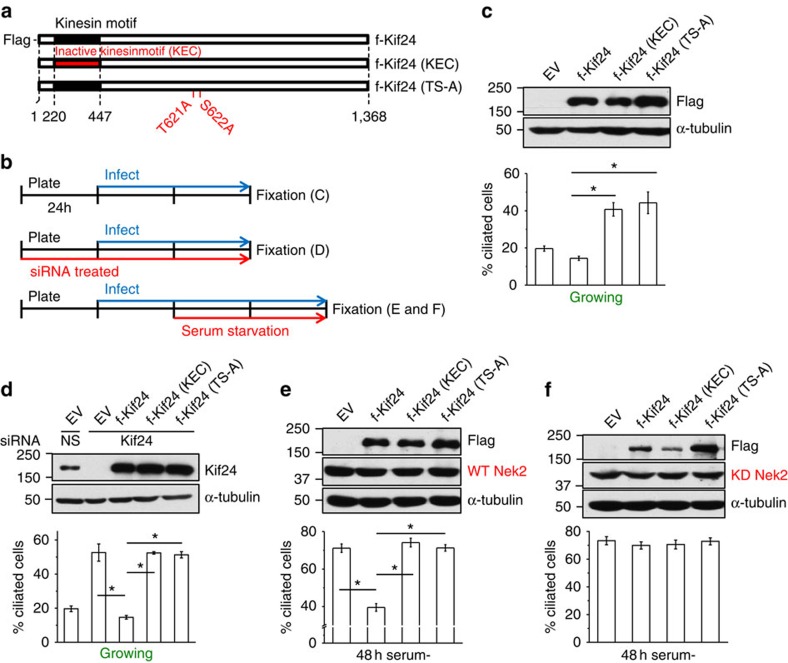
Identification of critical Nek2 phosphorylation sites in Kif24. (**a**,**b**) Schematic diagrams indicating Kif24 domains and point mutations (**a**) and experimental strategy (**b**). (**a**) Residues in red indicate phosphorylation sites regulated by Nek2, which have been mutated to prevent phosphorylation (T621A/S622A). (**b**) Diagram indicates the timing of viral infection, siRNA transfection, and/or serum starvation (each block represents 24 h). (**c**) Frequency of ciliation in growing RPE1 cells was measured after expression of EV (empty vector) control, WT Kif24, and the mutants described in panel (**a**). (**d**–**f**) Frequency of ciliation in RPE1 cells was examined after expression of indicated Flag-Kif24 mutants combined with depletion of endogenous Kif24 in growing RPE1 cells (**d**) or expression of WT (**e**) or kinase-inactive (KD) (**f**) Nek2 in serum-starved cells. Western blots of the resulting extracts were probed with antibodies against, Kif24, Flag and Nek2. α-tubulin was used as loading control. Data were obtained from three biologically independent experiments. Error bars show s.e.m. *P<0.05 (statistical significance tested against WT Kif24). Uncropped western blots are shown in Supplementary Fig. 13.

First, we addressed whether Nek2 and Kif24 coordinately regulate primary cilia formation through a common pathway. Consistent with our previous studies, we observed a dramatic increase in ciliation in cycling RPE1 cells depleted of Kif24, as compared with controls (Fig. 3a and Supplementary Fig. 4A). In addition, we found that depletion of Nek2 mimicked silencing of Kif24 (Fig. 3a and Supplementary Fig. 4A). However, co-silencing of Nek2 and Kif24 did not further increase the frequency of ciliation as compared with individual siRNA treatments, suggesting that they could work in a coordinated manner. Further, we stably expressed each protein (alone or together) in RPE1 cells and provoked quiescence through serum starvation. Since endogenous levels of Nek2 and Kif24 are negligible after serum starvation (Supplementary Fig. 3C), this allowed us to test the impact of altering the levels of each protein while limiting the expression of its partner (Fig. 3b). We found that ciliation was significantly diminished on expression of Nek2 or Kif24, and this effect was considerably more pronounced in cells co-expressing both proteins (Fig. 3b). The effect of Nek2 was dependent on its kinase activity, since expression of an inactive form of Nek2 (KD Nek2) overrode the effect of Kif24 expression (compare Fig. 4e,f and Supplementary Fig. 5D,E). Of note, cellular quiescence was not impaired in cells expressing Kif24 and/or Nek2, suggesting that reduced ciliation after ectopic expression did not result from cell cycle perturbations (Supplementary Fig. 4B). We next tested the impact of depleting Nek2 or Kif24 in cells ectopically expressing its partner. Remarkably, whereas Kif24 or Nek2 expression in cycling RPE1 cells showed significant reductions in ciliation as compared with controls, depletion of Nek2 or Kif24 in these cells, respectively, showed significant rescue in the frequency of ciliation, comparable to levels observed in populations lacking Kif24 or Nek2 (Fig. 3c and Supplementary Fig. 4C). Taken together, our results strongly suggest that Kif24 and Nek2 act in a common pathway to prevent cilium assembly.

Next, given their negative effect on cilium assembly, we asked whether combined expression of Kif24 and Nek2 could disassemble primary cilia in cells that had been brought to quiescence and allowed to ciliate (Supplementary Fig. 6). We found that ∼55% of cells were ciliated after 24 h of serum deprivation, and the percentage increased to ∼86 and 78% in the presence or absence of Kif24 and Nek2 expression, respectively (Supplementary Fig. 6B). Thus, Kif24 is unlikely to act on assembled cilia, despite proper localization at the base of the cilium in this setting (Supplementary Fig. 6C). These observations reinforce the conclusion that on activation by Nek2, Kif24 most likely acts to prevent assembly of nascent cilia and the nucleation of new cilia.

### Nek2 directly stimulates Kif24 activity by phosphorylation

We previously reported that Kif24 suppresses the aberrant growth of centriolar microtubules and primary cilia formation through its kinesin motif by showing that (1) abnormally long centrioles generated by Cep97 depletion in U2OS cells were abolished by expression of active Kif24, but not by a catalytically inactive (KEC) form, and (2) a purified fragment of Kif24 containing the kinesin motif alone was able to depolymerize microtubules *in vitro*^21^. Therefore, we tested whether Nek2 could regulate the microtubule-depolymerizing activity of Kif24 using analogous approaches. First, we found that depletion of Nek2 had minimal impact on centriolar elongation in U2OS cells, irrespective of expression of active or inactive Kif24 (Fig. 3d). As expected, Cep97 ablation led to aberrant elongation of centrioles in controls and cells expressing inactive Kif24, whereas expression of active Kif24 suppressed this phenotype (Fig. 3d and Supplementary Fig. 7A). Strikingly, however, ectopic expression of Kif24 did not rescue the centriolar phenotype in cells that were co-depleted of Nek2 and Cep97 (Fig. 3d).

Next, we tested the impact of Nek2-mediated phosphorylation of Kif24 on microtubule-depolymerization *in vitro*. Our attempts at expressing full-length Kif24 were unsuccessful, owing to the presence of an amino-terminal domain that destabilizes the protein^21^. Therefore, we expressed a flag-tagged Kif24 fragment lacking the first 92 amino acids (93–1,368) in insect cells, with or without Nek2 (Fig. 3e). We purified this recombinant Kif24, and in parallel, we treated protein obtained from cells that co-expressed Nek2 with λ phosphatase, which resulted in an increase in electrophoretic mobility, indicating that Kif24 was efficiently dephosphorylated (Fig. 3e). Next, we investigated whether Nek2-mediated phosphorylation could augment the microtubule-depolymerizing activity of Kif24. In agreement with our *in vivo* analysis, we observed a significant (>2 fold) increase in microtubule depolymerization when Kif24 was co-expressed with Nek2, as compared with Kif24 alone. However, this increase was eliminated with λ-phosphatase treatment. Moreover, we examined a recombinant fragment of Kif24 encompassing the kinesin domain that lacks putative Nek2 phosphorylation sites (Kif24 (93–547); Fig. 1c) and found that its activity was not augmented by Nek2 (Supplementary Fig. 7B). These data collectively suggest that Nek2-mediated phosphorylation of Kif24 facilitates its microtubule-depolymerizing activity *in vivo* and *in vitro*, thereby regulating ciliogenesis.

We next set out to identify the Nek2 phosphorylation site(s) that regulate Kif24 activity by systematically mutagenizing Kif24 and testing the impact of these mutants on cilium assembly. We examined cilium assembly in RPE1 cells expressing active (WT) or inactive (KEC) Kif24, as well as mutants that abrogated Kif24 phosphorylation (Fig. 4a and Supplementary Fig. 5A). We also expressed each protein in cells that were engineered to stably express active (WT) or inactive (KD) Nek2 and were serum starved for 48 h to determine their ability to suppress ciliation (Fig. 4b,e and f, and Supplementary Fig. 5D,E). In growing RPE1 cells, expression of KEC-Kif24 behaved as a dominant negative by significantly increasing ciliation (Fig. 4c and Supplementary Fig. 5B). Nek2 potently phosphorylates Kif24 residues within the regions spanning 548–678 and 671–825 (Fig. 1c). To delimit the residues required to stimulate Kif24 activity, we expressed a series of Kif24 internal deletion mutants and tested their impact on ciliation (Supplementary Fig. 5A). Notably, we found that certain Kif24 deletion mutants, encompassing residues 603–622 or 621–638 (Δ603–622, Δ621–638), but not others (Δ595–613), showed dominant negative effects on ciliation, similar to the KEC mutant, suggesting that a region spanning residues 603–638 contains a key regulatory phosphorylation site(s) responsible for Kif24 activation (Supplementary Fig. 5B). Next, we performed the converse experiment, examining whether the increase in ciliation provoked by Kif24 depletion in growing RPE1 cells can be rescued by expression of RNAi-resistant Kif24 mutants. We found that Kif24 (Δ595–613) rescued the depletion phenotype, whereas the activity of Kif24 (Δ621–638) and Kif24 (Δ603–622) was significantly compromised (Supplementary Fig. 5C). Furthermore, co-expression of Kif24 (Δ595–613) and WT-Nek2 in cells that were subsequently starved for 48 h resulted in a substantial decrease in ciliation, similar to expression of WT-Nek2 and Kif24, in sharp contrast with cells expressing WT-Nek2 alone, or WT-Nek2 and KEC-Kif24 (Supplementary Fig. 5D). Co-expression of KD-Nek2, on the other hand, abolished the impact of all active Kif24 deletion mutants on ciliation (Supplementary Fig. 5E), further confirming that Nek2 is indispensible for Kif24 function.

The internal deletions (Δ603–622 and Δ621–638) shared two overlapping, potential phospho-sites (T621/S622; Supplementary Fig. 5A), suggesting that these residues constitute regulatory targets of Nek2. Therefore, we generated a double point-mutated form of Kif24 (T621A/S622A, TS-A) in the GST-Kif24 fragment spanning from 585–645 and found that by *in vitro* kinase assay, the mutant exhibited reduced phosphorylation by Nek2 as compared with the WT fragment (Supplementary Fig. 8A). Next, we mutagenized full-length Kif24 to further determine whether these residues are required for Kif24 regulation. Using WT Kif24 and Kif24 (KEC) as positive and negative controls, respectively, we determined whether Kif24 (TS-A) has a dominant negative effect by altering ciliary induction in growing cells with or without depletion of Kif24 (Fig. 4c,d) and whether it can impact ciliation in the presence of active or inactive Nek2 in serum-starved cells (Fig. 4e,f). Conversely, we also created phospho-mimetic mutants in which we converted both residues to aspartic acid (T621D/S622D). We found that these mutations did not mimic the phosphorylated state, as they did not result in constitutive activation of Kif24 in the absence of Nek2, and therefore, we did not further pursue the use of this mutant (Supplementary Fig. 9). Remarkably, however, we found that Kif24 (TS-A) largely nullified Kif24 activity in each of these assays, even in the presence of exogenous WT-Nek2 (Fig. 4). Furthermore, we compared the sub-cellular localization of both WT Kif24 and Kif24 (TS-A) during the G0, G1, S and G2 phases of the cell cycle and found that they were similar, suggesting that the loss of activity in the Kif24 (TS-A) mutant is not due to aberrant localization (Supplementary Fig. 10). We noted that other Kif24 residues appeared to be robustly phosphorylated by Nek2 *in vitro*, including a region spanning residues 671–825 (Fig. 1c), which prompted us to create two Ser-to-Ala mutations within this fragment (S789A and S810A). We found that the S789A mutation led to a sharp decline in signal, suggesting that this residue is phosphorylated by Nek2 (Supplementary Fig. 8B). However, the S789A mutation did not significantly alter the extent of ciliation when combined with Δ595–638 (Supplementary Fig. 8C), further confirming that residues T621 and S622 of Kif24 constitute specific, critical regulatory targets of Nek2.

### Phosphorylation leads to conformational change of Kif24

**Figure 5:**
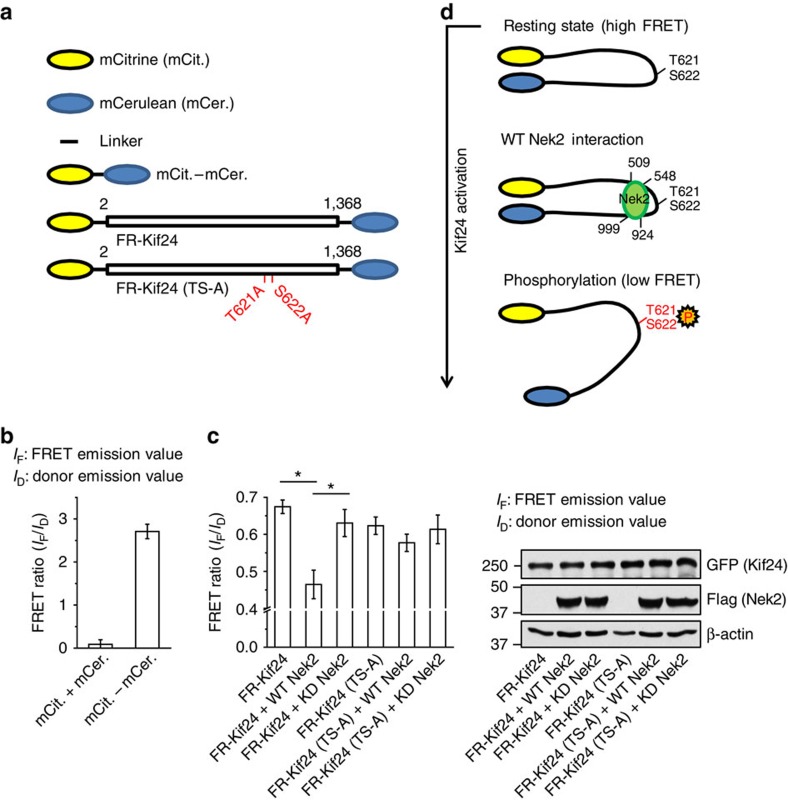
Nek2-dependent phosphorylation induces conformational change in Kif24. (**a**) Schematics of constructs used for FRET assay. (**b**,**c**) FRET signals normalized to donor emissions (*I*_F_/*I*_D_, FRET ratio) of the indicated samples. Co-expressed mCitrine and mCerulean (mCit.+mCer.) was used as negative control and tethered mCitrine–mCerulean (mCit.−mCer.) as a positive control displaying high FRET. Expression of probes and Nek2 in panel (**c**) was confirmed by immunoblotting with anti-GFP antibodies to detect FRET probes (FR-Kif24 and FR-Kif24 (TS-A)) or anti-Flag antibodies to detect tagged Nek2. β-actin was used as a loading control. Data were obtained from three independent experiments. Error bars show s.e.m. *P<0.05. (**d**) Model of Kif24 activation by Nek2.

Kif24 is a member of the Kinesin-13 family, and it has been shown that phosphorylation of other family members alters their activity^21^,^25^,^26^,^27^,^28^. For example, the mitotic kinase, Plk1, has been shown to phosphorylate Kif2A, B and C, promoting their microtubule-depolymerizing activity^25^,^26^,^27^. More recently, it has been shown that phosphorylation of Kif2C/MCAK by Aurora B induces a conformational change that alters the intra-molecular association between the amino- and carboxy-terminal domains, switching it from a closed to an open state with reduced catalytic activity^28^. Of note, we found that the amino- and carboxy-terminal fragments of Kif24 are able to interact (Supplementary Fig. 11A), and this result prompted us to test whether Kif24 could also undergo a phosphorylation-dependent conformational change using a Kif24 Förster resonance energy transfer (FRET) (FR-Kif24) probe with an amino-terminal mCitrine acceptor fluorophore and a carboxy-terminal mCerulean donor fluorophore (Fig. 5a). The probes were expressed in HEK293 cells, and the efficiency of energy transfer between the donor and acceptor fluorophores was measured. First, as expected, we observed low FRET in cells that co-expressed soluble mCerulean and mCitrine, whereas tethering of mCitrine to mCerulean produced strong FRET (Fig. 5a,b). Next, we tested whether Nek2 altered the conformation of Kif24 by co-expressing the FR-Kif24 probe with functional Nek2 (WT Nek2). We observed a significant decrease in the FRET signal in cells that expressed Nek2, whereas co-expression of FR-Kif24 with catalytically inactive Nek2 (KD Nek2) caused no significant change in FRET (Fig. 5c). We further created a mutant version of FR-Kif24 in which sites phosphorylated by Nek2 were changed to alanine (FR-Kif24 (TS-A)). This probe exhibited similar FRET levels to the WT probe, but unlike the WT probe, co-expression of WT Nek2 did not alter the FRET signal (Fig. 5c). Furthermore, we found that the FRET of FR-Kif24 increased significantly in cells deprived of endogenous Nek2 (Supplementary Fig. 11B). Taken together with our observation that the amino- and carboxy-terminal regions of Kif24 can interact, these data suggest that Kif24 undergoes a conformational change in response to Nek2-mediated phosphorylation at T621/S622, which results in an ‘open’ conformation in which the amino- and carboxy- termini are separated (Fig. 5d). These findings are reminiscent of a ‘tail inhibition’ model, wherein the interaction between the head (containing the motor domain) and tail motifs in a cohort of myosin and kinesin motor proteins leads to an auto-inhibitory conformation that is reversed in response to activating signals^29^,^30^.

### Activated Kif24 disassembles cilium during G2/M phases

Although primary cilia are known to assemble in a fraction of proliferating RPE1 cells, ciliogenesis is highly induced as cells enter quiescence, and the organelle disassembles when quiescent cells are exposed to growth factors^1^,^2^,^3^,^4^. Cilium resorption has been shown to occur in two stages: the first stage occurs immediately after serum re-stimulation, followed by a robust wave of resorption at the G2/M transition^1^,^2^,^3^,^4^. It is attractive to posit that ensuring cilium resorption during the G2/M phase constitutes an additional fail-safe barrier, as this organelle is incompatible with a mitotic spindle. Cilium disassembly is triggered by a complex mechanism involving activation of Aurora A, which stimulates HDAC6-mediated deacetylation and destabilization of axonemal microtubules, leading to cilium resorption^3^. Since the Nek2–Kif24 pathway negatively regulates ciliogenesis by actively depolymerizing microtubules, we asked whether it is linked to the activation of Aurora A and HDAC6.

**Figure 6:**
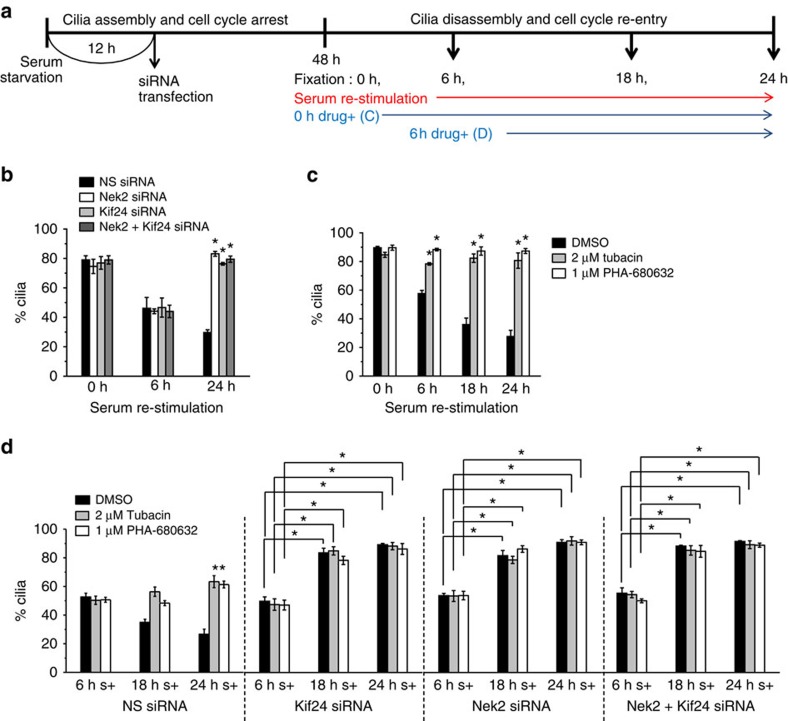
The role of Kif24 can be distinguished from the Aurora A-HDAC6 pathway during primary cilium disassembly. (**a**) Schematic illustration of experimental strategy. (**b**–**d**) RPE1 cells were transfected with siRNAs or treated with inhibitors during the indicated periods, and ciliation was assessed at each indicated time points. Treatments with inhibitors of HDAC6 (Tubacin) and Aurora A (PHA-680632) were performed as indicated. Cells were collected at the indicated times after serum re-stimulation. Data were obtained from three biologically independent experiments. Error bars show s.e.m. *P<0.05. Statistical significance was tested against NS siRNA at 24 h (**b**), dimethylsulphoxide treatment at 6, 12 and 24 h (**c**), and at 6 h serum re-stimulated time point for each drug treatment (**d**).

First, we examined the expression profiles of Nek2 and Kif24 at various times after serum re-stimulation of quiescent cells. We found that Nek2 and Kif24 were expressed from 18 h onward, whereas they were not detectable at earlier time points that we examined (Supplementary Fig. 3C), suggesting that Nek2–Kif24 could play a role in the second ‘phase’ of ciliary disassembly during the later stages of the cell cycle^2^,^3^,^20^. Next, we ablated Nek2 and/or Kif24 at 12 h post-serum deprivation (to avoid the consequences of eliminating Nek2 and/or Kif24 in growing cells, which would promote premature ciliation) and visualized cilia after 48 h of serum starvation (0 h time point), and 6, 18 and 24 h post-serum stimulation (Fig. 6a). We compared the kinetics of this response to cells treated with specific inhibitors of Aurora A (PHA-680632) and HDAC6 (Tubacin) at each time point (Fig. 6a). In accordance with the expression profiles of Nek2 and Kif24, depletion of Nek2 and/or Kif24 had no effect on ciliation after 6 h re-stimulation (Fig. 6b). Interestingly, however, we observed dramatic assembly of primary cilia at a later time point (24 h) in cells lacking Nek2 and/or Kif24, which likely reflects *de novo* cilium assembly and/or possible re-growth of cilia that may have previously commenced or completed the disassembly process (Fig. 6b). This suggests that cilium assembly and disassembly co-exist in a dynamic equilibrium in which the concerted action of Nek2 and Kif24 can prevent mother centrioles/basal bodies from nucleating a new cilium.

Further, we could distinguish the activities of Nek2–Kif24 from the Aurora A-HDAC6 pathway, since treatment of serum re-stimulated cells with either Aurora A or HDAC6 inhibitors at 0 h clearly prevented cilia disassembly as compared with the dimethylsulphoxide control (Fig. 6c)^3^,^31^. We reasoned that overlapping roles of these two pathways might prevent us from detecting any differences in ciliation if both pathways were ablated. Therefore, to distinguish the role of Nek2–Kif24 in cilia disassembly from the Aurora A-HDAC6 pathway, we depleted Kif24 and/or Nek2 from serum-starved cells and treated them with Tubacin or PHA-680632, from 6 h post-re-stimulation (Fig. 6a). In contrast with the consistent level of ciliation observed in the drug-treated, control siRNA-treated cells, an increase in cilia formation was observed in Nek2- and/or Kif24-depleted cells, which persisted throughout drug treatment at 18 and 24 h post-serum stimulation (Fig. 6d). This suggests that Nek2–Kif24 and Aurora A-HDAC6 play distinct, sequential roles during cilia disassembly as cells re-enter the cell cycle from quiescence: Aurora A-HDAC6-mediated axonemal disassembly is succeeded by Nek2–Kif24-mediated suppression of nascent cilium assembly and, potentially, ciliary re-assembly in cells that have already disassembled their cilia. In this sense, Kif24 activity could ensure the completion of cilium removal in the later stages of the cell cycle.

### A potential role for Nek2 and Kif24 in mammary tumorigenesis

It has recently been shown that Nek2 is a proto-oncogene that is highly expressed in various cancer types, including breast cancer^13^,^14^,^15^. Furthermore, it is known that mammary epithelial cells lose primary cilia during the process of oncogenic transformation^8^,^10^. Therefore, we compared the expression levels of Kif24 and Nek2 in a panel of breast cancer cell lines with the normal human mammary epithelial cell line (HMEC) as control. By normalizing the expression profiles of Kif24 and Nek2 with HMECs, we found that Kif24 protein levels were comparable or modestly elevated across the panel of cell lines, whereas Nek2 was strongly upregulated (∼5–30-fold) in all breast cancer cell lines that we examined (Supplementary Fig. 12A–C). Although they are non-tumorigenic, MCF10A are immortal and hyperplastic, and these cells also exhibited enhanced expression of Nek2 (refs 10, 11). This led us to hypothesize that in hyper-proliferative mammary and breast cancer cells, the uncontrolled overexpression of Nek2 could aberrantly augment Kif24 activity, suppressing cilia formation and facilitating proliferation, a hallmark of breast tumours^8^,^10^.

**Figure 7:**
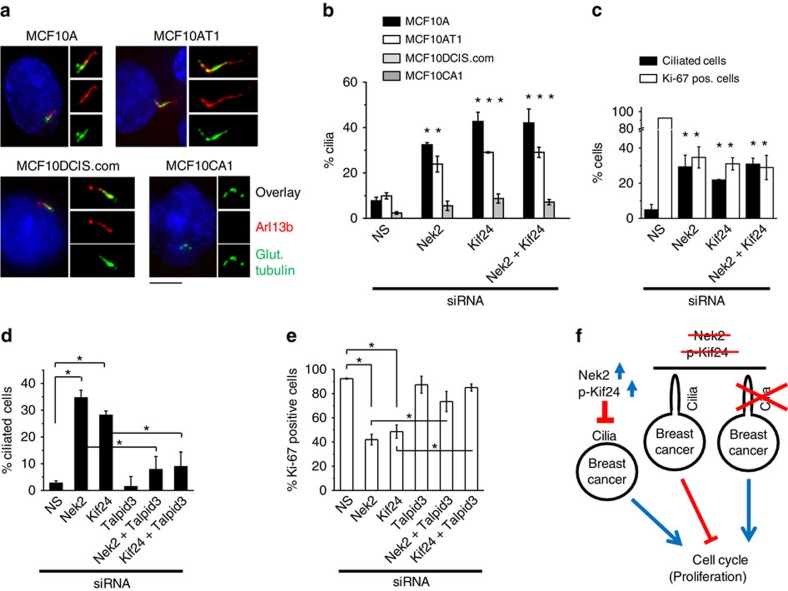
Removal of Nek2 or Kif24 restores ciliation in breast cancer cell lines. (**a**) Immunofluorescence detection of primary cilia, visualized with antibodies against glutamylated tubulin (GT335) and Arl13B, in the MCF10 cell line series used in our study. Scale bar: 5μm. (**b**) Depletion of Nek2 or Kif24 restores ciliation in a subset of MCF10 cell lines. (**c**) Proliferation (Ki-67 positivity) and ciliation were assessed in Hs578T cells depleted of Nek2 and/or Kif24. Primary cilia (**d**) and proliferation (Ki-67 positivity) (**e**) were counted after RNAi-mediated depletion of Kif24 or Nek2 in combination with Talpid3 (as indicated) in Hs578T cells. (**f**) Summary of our findings in breast cancer cells. Data were obtained from three biologically independent experiments. Statistical significance was tested against NS siRNA for each cell line (**b**) and for each parameter tested (ciliation, black bar or Ki-67 positivity, white bar) (**c**). For panels (**d**) and (**e**), statistical analyses were performed by comparing samples indicated by brackets: NS versus Nek2 or Kif24 siRNA-treated cells, Nek2 versus Nek2+Talpid3 siRNA-treated cells, and between Kif24 and Kif24+Talpid3 siRNA-treated samples (**d**,**e**). Error bars show s.e.m. *P<0.05.

To test whether blocking Nek2 or Kif24 can rescue ciliogenesis in breast cancer cells, we took advantage of a series of cell lines, derived from spontaneously immortalized MCF10A cells, that have been transformed with oncogenic *ras* (V12G) and clonally selected after passage in mice^32^,^33^,^34^. The resulting lines—MCF10AT (pre-malignant mammary cells), MCF10DCIS.com (comedo-type ductal carcinoma *in situ*), and MCF10CA1 (invasive, metastatic carcinoma)—represent increasing grades of malignancy^32^,^33^,^34^. As reported, we observed a gradual decrease in the frequency of ciliation with increasing malignancy, as determined by ciliary marker (Arl13b and GT335) staining, and MCF10CA1 were completely devoid of cilia (Fig. 7a,b)^10^. Remarkably, ablation of Nek2 or Kif24 in growing cells led to a significant increase in ciliation of MCF10A, AT1 and DCIS.com cells (Fig. 7b). However, there was no impact of depletion on the most invasive cell line, CA1, suggesting the possible accumulation of additional oncogenic ‘hits’. Interestingly, co-depletion of Nek2 and Kif24 did not have an additive effect on ciliation, suggesting that Nek2 and Kif24 function in concert (Fig. 7b). Moreover, we observed dramatically enhanced expression of Nek2 in the DCIS.com and CA1 cell lines and upregulation of Kif24 in the CA1 cell line, as compared with the MCF10A and AT1 lines (Supplementary Fig. 12D). These data lend further credence to the idea that Nek2 and Kif24 could have proto-oncogenic tendencies in malignant breast cancer cells.

In addition, we tested a triple-negative breast cancer cell line of the basal subtype derived from human patients (Hs578T) as a second, physiologically relevant model to determine whether ciliation can be restored by modulating the Nek2–Kif24 pathway^35^. We reasoned that restricting proliferation through restoration of primary cilia could provide a clinically useful tumour suppressive mechanism. We found that RNAi-mediated depletion of Kif24 and/or Nek2 led to a significant increase in ciliation and a corresponding decrease in Ki-67 expression (Fig. 7c and Supplementary Fig. 12E). To determine whether this increase in quiescence is induced by ciliation, we co-depleted Nek2 or Kif24 and Talpid3, a protein essential for cilium assembly^36^. We found that Talpid3 depletion overrode the ability of these cells to ciliate on Nek2 or Kif24 knockdown (Fig.7d and Supplementary Fig. 12E). Importantly, we found that Nek2- or Kif24-depleted cells devoid of primary cilia exhibited increased Ki-67 expression as compared with the corresponding ciliated cells (Fig. 7e), strongly suggesting that aberrant activation of the Nek2–Kif24 pathway promotes cilium disassembly and proliferation. Furthermore, abrogating this defective Nek2/Kif24 activation can restore primary cilia formation and restrict proliferation in breast cancer cells (Fig. 7f).

## Discussion

Nek2 plays an established role in centrosome separation, and a number of centrosomal substrates have been identified. Nek2 has also been shown to negatively regulate primary cilia assembly, since ablation of the kinase led to an increase in cilia assembly and a delay in disassembly^20^. Despite these observations, mechanistic insights and substrates were lacking. Here we identify Kif24 as a major substrate of Nek2 in the regulation of primary cilia formation. Further, we have shown that Nek2 phosphorylation induces a conformational change that stimulates the ability of Kif24 to depolymerize microtubules. Nek2 and Kif24 co-localize at the distal ends of mother centrioles in S/G2 phase, consistent with a role in ensuring the disassembly of a ciliary axoneme, which is incompatible with a mitotic spindle in mammalian cells^37^,^38^,^39^,^40^. Proteins related to Kif24 and Nek2 are found in diverse ciliated and flagellated species, including *Chlamydomonas, Tetrahymena*, trypanosomes and *Giardia*, wherein orthologs of Kif24 (Kinesin-13) and Nek2 (NIMA-related kinases) have been implicated in flagellar assembly and disassembly^41^,^42^,^43^. Intriguingly, *Chlamydomonas* Kinesin-13 protein (CrKinesin 13) functions in both processes, and it is targeted to the disassembling flagella and is phosphorylated as new flagella are assembled^44^,^45^. In addition, two NIMA-related kinases, FA2 and CNK2, have been shown to regulate flagella assembly and disassembly in *Chlamydomonas*^42^,^46^. CNK2 localizes to the axoneme, where it can modulate flagellar length by altering the rate of axoneme disassembly^42^. Likewise, overproduction of a *Tetrahymena* kinase related to CNK2 reduces cilia length^43^. Moreover, it has recently been reported that a member of the mammalian Kinesin-13 family, Kif2A is phosphorylated by Plk1 at the sub-distal appendage of the basal body to facilitate primary cilia disassembly shortly (within 4 h) after serum stimulation of quiescent cells^47^. Thus, although there appear to be both mechanistic similarities and differences across species, these findings suggest a widespread, evolutionarily conserved role for Kinesin-13 and Nek2 orthologs in ciliary and flagellar assembly/disassembly.

We showed that there are two regions of Kif24 (a middle and carboxy-terminal domain) required for interaction with Nek2 (Fig. 5d and Supplementary Fig. 1). Further, our structure–function and mechanistic studies revealed that Kif24 exhibits intra-molecular interactions, which promote a ‘closed’, auto-inhibited state, properties reminiscent of a group of kinesin and myosin motor proteins^29^,^30^. Moreover, analogous to other kinesins, we found that binding by Nek2 and activating phosphorylation promotes an ‘open’ conformation of Kif24. In contrast, others have shown that Aurora B-mediated phosphorylation induced a less active, ‘open’ conformation of Kif2C/MCAK (ref. 28), suggesting inherent differences between the members of the Kinesin-13 family. In future studies, it will be interesting to dissect the molecular determinants that confer such distinct properties on Kif2C/MCAK and Kif24.

Our Kif24 ablation data have two important implications for our understanding of cilium assembly and disassembly during the cell cycle. First, our experiments suggest that conditions are permissive to cilium assembly throughout interphase, and that elimination of certain barriers, enforced by Nek2 and its substrate, Kif24, and possibly other proteins, unveils this permissivity. Even as late as S and G2 phase, assembly and disassembly may be in equilibrium, but the equilibrium is shifted towards the unciliated state through the timely expression of Nek2 and Kif24 during this period of the cell cycle. Although we favour a model in which Nek2 and Kif24 prevent re-growth of cilia that have previously resorbed as well as *de novo* assembly of the organelle, we have not distinguished between these possibilities in our studies, and future experiments will be required to determine whether this is indeed the case. Second, our data suggest that Kif24 function, coupled to Nek2 activity, provides a counterpart to the Aurora A-HDAC6 pathway of cilium disassembly initiated during cell cycle re-entry. An analogous mechanism may operate in *Chlamydomonas*, wherein the Nek2-related kinase, CNK2, supports a feedback control mechanism to maintain the balance between the assembly and disassembly of the axoneme, and the protein induces the disassembly process^48^. Similarly, Nek2 in mammalian cells phosphorylates Kif24 to establish a fail-safe mechanism by preventing ciliogenesis, thereby ensuring the timely resorption of cilia before mitosis. By analogy to the famous tale in Greek mythology in which Hercules attempted to slay the multi-headed Hydra able to re-grow its severed heads—the effort only succeeded with the help of his nephew, Iolaus, who cauterized each neck after decapitation to prevent ciliogenesis—a team-effort involving Hercules (Aurora A-HDAC6) and Iolaus (Nek2–Kif24) may be required to ensure the loss of cilia before mitosis.

Our studies on Kif24 and Nek2 also provide potentially useful clinical avenues of exploration. Nek2 was recently identified as an oncogene, and its expression was found to be upregulated in numerous tumour types, including breast tumours^13^,^14^,^15^. For example, a approximately ninefold increase in the per cent of cells with ‘strong’ Nek2 protein staining was observed in tumours, as compared with normal breast tissue, and its upregulation was closely associated with poor prognosis and high recurrence rates in patients^13^,^14^. These findings are consistent with our observation that Nek2 proteins levels are highly upregulated in breast cancer cell lines (Supplementary Fig. 12A–C)^13^,^14^,^15^. Our data implicate Kif24 as a physiological substrate of Nek2, among the few that have been identified^19^,^49^,^50^,^51^. While Nek2 is likely to have many substrates relevant to growth control, we have identified Kif24 as an important target, and our studies suggest that either could be rate-limiting for proliferation in certain circumstances. Both Kif24 and Nek2 are enzymes, suggesting that they are potentially druggable. Indeed, progress has been made in developing inhibitors of Nek2 (ref. 52). Our data suggest the utility of inhibiting their function in human tumours. Importantly, our experiments with the MCF10 cell line model suggest a means for therapeutic intervention, although our results strongly imply that such intervention must occur in the early stages of progression of the tumour to ensure efficacy. Clearly, tumours in the early stages of the disease retain the capacity to ciliate^8^, and strategies directed at these populations are therefore more likely to succeed. Future studies in other tumour types will be required to explore the generality of this therapeutic strategy.

## Methods

### Cell culture and plasmids

Human RPE1-hTERT, U2OS and HEK293 cells were obtained from the American Type Culture Collection and grown in Dulbecco's Modified Eagle Medium (DMEM) supplemented with 10% foetal bovine serum (FBS). The human breast cancer cell lines MCF7, ZR57-1, MDA-MB-231, MDA-MB-468, Hs578T and MCF10A were obtained from American Type Culture Collection, and the HMEC cell line was obtained from Lonza. The cell lines engineered to express oncogenic H-ras, MCF10AT and MCF10CA1, were obtained from Karmanos Cancer Institute, and MCF10DCIS.com was obtained from Wayne State University. HMEC cells were cultured in MEGM SingleQuot Kit Supplement & Growth Factors. MCF7, MDA-MB-231, MDA-MB-468, Hs578T, MCF10DCIS.com and MCF10CA1 were maintained in DMEM supplemented with 10% FBS, ZR57-1 was cultured in RPMI-1640 supplemented with 10% FBS, and MCF10A and MCF10AT1 cell lines were maintained in DMEM/F-12 supplemented with 0.1 μg ml^−1^ cholera toxin, 10 μg ml^−1^ insulin, 0.5 μg ml^−1^ hydrocortisone, 0.02 μg ml^−1^ epidermal growth factor (EGF) and 5% horse serum. The MCF10A, AT1, DCIS.com and CA1 cell lines were cultured for 7 days in growth media to reach confluence to maximize the induction of ciliation^10^. Sf9 and High Five cells were grown in Grace’s insect medium supplemented with 10% FBS at 27 °C.

To generate a Flag-tagged or GFP-tagged version of Kif24, human Kif24 cDNA was sub-cloned into pLVX-3xflag-IRES-puromycin or pLVX-GFP-IRES-puromycin vector, respectively. To generate Kif24 mutants, Kif24 fragments encoding amino-acid residues 924–1,368 and 1–923, or full-length Kif24 with internal deletion mutants of amino-acid residues 595–613, 621–638 and 603–622 were amplified by PCR and sub-cloned into pLVX-3xflag-IRES-puromycin or pLVX-GFP-IRES-puromycin vector. Human Nek2A cDNA was obtained from Dr Kunsoo Rhee (Seoul National University, South Korea) and sub-cloned into pLVX-3xflag-IRES-puromycin or pLVX-GFP-IRES-puromycin vector to generate flag-Nek2 or GFP–Nek2, respectively. To generate recombinant GST-Kif24, fragments of Kif24 encoding amino acids 1–218, 213–547, 548–678, 671–825, 813–941, 935–1,078, 1,069–1,227, 1,213–1,368, 548–678, 548–594, 585–645, 639–678, 671–825, 671–733, 724–788 and 752–825 were amplified by PCR and sub-cloned into pGEX-6 P-1 vector. To generate recombinant flag-Kif24, a Kif24 fragment spanning residues 93–1,368 was PCR amplified and sub-cloned into pFastBAC vector. To generate FRET reporters, the mCerulean and mCistrine-containing MCAK-ΔNT and MCAK-ΔCT were amplified by PCR and sub-cloned into pEGFP-C1 vector, substituting the EGFP fragment. mCerulean alone, mCitrine alone, mCitrine–mCerulean, Kif24 and Kif24 (TS-A) were also sub-cloned identically to the modified pEGFP-C1 vector.

Pfu Turbo (Stratagene) and Phusion HF (NEB) polymerases were used for all PCR reactions. All constructs were verified by DNA sequencing. Expression of WT and mutant Kif24 or Nek2 in RPE1 and U2OS cell lines was carried out through lentivirus infection. Transient transfections of plasmids into HEK293 cells were performed using polyethyleneimine according to the manufacturer’s instruction.

### Immunofluorescence microscopy

Indirect immunofluorescence was performed by fixing the cells with ice-cold methanol for 5 min on ice or with 10% formalin solution (Sigma-Aldrich) for 15 min at room temperature, and permeabilization with 0.5 or 1% Triton X-100, diluted in PBS, for 5 min at room temperature. Slides were blocked with 3% bovine serum albumin (BSA), diluted in PBS, before incubation with primary antibodies. Secondary antibodies used were Cy3- (Jackson Immunolabs) or AlexaFluor488- (Invitrogen) conjugated donkey anti-mouse or anti-rabbit IgG. Cells were then stained with 4,6-diamidino-2-phenylindole (DAPI), and slides were mounted using Prolong Gold anti-fade reagent (Life Technologies). Slides were observed and photographed using a Nikon Eclipse E800 microscope (Nikon; × 63 or × 100, numerical aperture (NA) 1.4) equipped with a Photometrics Coolsnap HQ CCD camera. Images were acquired and processed with MetaMorph7 (Molecular Devices). Percentage of cells with distal Nek2 puncta, cilia, elongated centrioles and Ki-67 staining was quantified by first counting the total number of cells using 4,6-diamidino-2-phenylindole from a randomly selected field, and within the same field, switching filters to visualize and count the cells expressing the marker of interest. Minimally, ten random fields were selected to generate a percentile.

### Antibodies

Antibodies used in this study include polyclonal rabbit anti-Kif24 (ref. 21) (1:500 for western blot (WB) and 1:200 for immunofluorescence (IF)), anti-Nek2 (1:100 for WB and IF, cat. #610593, BD Biosciences), anti-Cep164 (1:2,000 for IF, gift from Dr Eva Lee and 1:5,000 for IF, cat. #45330002, Novus), anti-centrin-2 (1:1,000 for IF, cat. #04-1624, Millipore), β-actin (1:5,000 for WB, cat. #ab6276, Abcam), anti-α-tubulin (1:5,000 for WB, cat. #T5168), Flag (1:1,000 for WB and 1:200 for IF, cat. #F1804) and γ-tubulin (1:1,000 for IF, cat. #T6557, all from Sigma-Aldrich), anti-GFP (1:1,000 for WB, cat. #G1544, Sigma-Aldrich and 1:100 for WB, cat. #sc-8334, Santa Cruz Biotechnology), anti-glutamylated tubulin (GT335) (1:1,000 for IF, cat. #AG-20B-0020-C100, Adipogen), anti-IFT88 (1:500 for IF, cat. #13967-1-AP, Proteintech), anti-CPAP (1:500 for IF, gift from Dr Tang Tang), anti-Talpid3 (ref. 36) (1:500 for WB), anti-SAS6 (1:100 for IF, cat. #sc-81431), and Cyclin A (1:100 for WB, cat. #sc-596, all from Santa Cruz Biotechnology), anti-phospho-ser10-Histone H3 (1:1,000 for WB, cat. #06-570, Millipore), anti-Arl13b (1:100 for IF, gift from T. Katada and K. Kontani), and anti-Ki67 (1:1,000 for IF, cat. #ab15580, Abcam).

### RNAi

Synthetic siRNA oligonucleotides were obtained from Dharmacon (GE Healthcare). Transfection of siRNAs using Lipofectamine RNAiMAX (Invitrogen) was performed according to the manufacturer’s instructions. The 21-nucleotide siRNA sequences for the non-specific control, Cep97 and Kif24 were described previously^36^. The 21-nucleotide siRNA sequence for human Nek2 was 5′-GATGCAATTTGGTCATTAATT-3′ and 5′-GAAAGGCAATACTTAGATGTT-3′.

### Immunoprecipitation

Cells were lysed with E1A lysis buffer (ELB) buffer (50 mM Hepes pH 7, 250 mM NaCl, 5 mM EDTA/pH 8, 0.1% NP-40, 1 mM dithiothreitol (DTT), 0.5 mM 4-(2-aminoethyl)benzenesulfonyl fluoride hydrochloride (AEBSF), 2 μg ml^−1^ leupeptin, 2 μg ml^−1^ aprotinin, 10 mM NaF, 50 mM ß-glycerophosphate and 10% glycerol) at 4 °C for 30 min. For immunoprecipitation, 2–5 mg of the supernatant after centrifugation of lysate was incubated with an appropriate antibody at 4 °C for 1 h followed by protein A-sepharose incubation for an additional 2 h. The resin was washed with ELB buffer, and the bound polypeptides were analysed by SDS-PAGE and immunoblotting. An amount of 50 μg of lysate was typically loaded in the input lane. For Flag-Kif24 immunoprecipitations, polyethyleneimine-transfected HEK293 cells were collected 48–72 h after transfection. Flag-M2 beads (cat. #A2220, Sigma-Aldrich) were incubated with cell extract at 4 °C for 3 h and subsequently washed and processed as described above.

### Recombinant proteins

Baculovirus expressing GST-Kif24 (93–547) and f–Kif24 (93–1,368) were generated with the Bac-to-Bac baculovirus Expression System (Invitrogen). Baculovirus expressing Myc-Nek2 was a gift from Dr Eva Lee (University of California at Irvine). Virus-infected Sf9 or High Five cells were lysed with lysis buffer (50 mM Tris pH 7.5, 100 mM NaCl, 1 mM MgCl_2_, 1% Triton X-100, 1 mM DTT, 0.5 mM AEBSF, 2 μg ml^−1^ leupeptin and 2 μg aprotinin) and incubated with glutathione agarose (Sigma-Aldrich) or FLAG-M2 beads (Sigma-Aldrich). GST- and flag-tagged recombinant proteins were eluted with 20 mM glutathione or 1 mg ml^−1^ flag peptide (Sigma-Aldrich) in elution buffer (100 mM Tris pH8, 50 mM NaCl, 1 mM MgCl_2_, 1 mM DTT and 0.1% Triton X-100) and dialyzed overnight in 4 °C into dialysis buffer (80 mM piperazine-N, N'-bis (2-ethanesulfonic acid) (PIPES) pH 6.9, 1 mM ethylene glycol tetraacetic acid, 1 mM MgCl_2_, 50 mM NaCl, and 1 mM DTT). Phosphorylated Kif24 (93–1,368) and Kif24 (93–547) were generated by co-infecting Sf9 or High Five cells with Kif24 and Nek2 baculoviruses. Lambda phosphatase (NEB) treatments were performed according to the manufacturer’s instructions.

Flag-tagged WT Nek2 and KD Nek2 used in kinase assays were expressed in HEK293 cells, lysed in ELB buffer, and incubated with anti-Flag (M2) agarose beads (Sigma) for 2 h at 4 °C. Beads were washed twice with the ELB buffer containing 500 mM NaCl. Purified proteins were eluted with 1 mg ml^−1^ Flag peptide (Sigma-Aldrich) for 30 min and dialyzed into dialysis buffer.

### *In vitro* kinase assay

GST-fragments of Kif24 were co-incubated with active (f–WT#Nek2) or inactive (f–KD#Nek2) Nek2 in kinase assay buffer (50 mM Hepes pH 7, 10 mM MgCl_2_, 5 mM MnCl_2_, 1 mM NaF, 4 mM beta-glycerol phosphate, 10% glycerol, 1 mM DTT and 5 μM ATP) containing 5 μCi γ-[^32^P]ATP for 30 min at 30 °C. Casein was used as a positive control, and GST alone was a negative control.

### Microtubule-depolymerization assay

For the microtubule-depolymerization assay, recombinant Kif24 was incubated with 2 mM ATP and 4 μM Taxol-stabilized microtubules from porcine brain (cat. #T240-A, Cytoskeleton) in reaction buffer (80 mM PIPES (pH 6.9), 1 mM ethylene glycol tetraacetic acid, 2 mM MgCl_2_, 50 mM NaCl, 1 mM DTT and 2 μM Taxol) for 30 min at room temperature and was subjected to ultracentrifugation at 100,000*g* for 15 min at 25 °C. Supernatant and pellet fractions were subjected to SDS-PAGE and stained with Coomassie blue.

### Förster resonance energy transfer

For FRET analysis, the cyan fluorescent protein mCerulean was used as the donor fluorophore, and the yellow fluorescent protein mCitrine was used as the acceptor. HEK293 cells were transfected with plasmids encoding fluorescent proteins, FRET probes and Nek2 using Lipofectamine 3000 (Life Technologies). Kif24 FRET probes and Nek2 plasmids were mixed together in a 1:3 ratio to ensure that cells expressing the probe also expressed Nek2. For co-transfection of siRNA and FRET-Kif24, HEK293 cells were transfected with NS or Nek2 siRNA using RNAiMAX, 24 h before Lipofectamine 3000-transfection of FRET-Kif24. FRET measurements were made on cells from three independent transfections, from each of which more than 100 cells were analysed. Images were captured using an Applied Precision PersonalDV live-cell imaging system. The FRET reporters were excited at 430 nm and the CFP/YFP emission passed through a DeltaVision Live Filter set (CFP and YFP) to obtain separate mCerulean and mCitrine images using a widefield fluorescence inverted microscope (× 20/0.75, Olympus IX-71) equipped with a Photometrics CoolSNAP HQ2 CCD camera. Images were acquired using SoftWoRx suite software, and the mean fluorescence intensity was measured from each ROI using ImageJ to calculate the FRET ratio. In a majority of cells, Kif24 probes were localized to puncta that likely correspond to microtubule ends. Regions of interest from these cells were selected for FRET measurements. Cells in which the probes were expressed in large aggregates or uniformly at high levels were excluded from the analysis.

FRET signals were calculated and normalized to donor emissions using the following formula: *I*_F_/*I*_D_=(FRET−*α*DONOR−*β*ACCEPTOR)/DONOR where all fluorescence signals were corrected for background fluorescence, *α* is the fraction of mCerulean donor signal detected in the FRET channel and *β* is the fraction of a mCitrine acceptor signal seen in the FRET channel after excitation with 430 nm light. For the microscope used in our studies, we determined that *α*=0.96 and *β*=0.15.

### Statistical analysis

The statistical significance of the difference between two means was determined using a two-tailed Student’s *t*-test. Differences were considered significant when *P*<0.05.

## Additional information

**How to cite this article:** Kim, S. *et al.* Nek2 activation of Kif24 ensures cilium disassembly during the cell cycle. *Nat. Commun.* 6:8087 doi: 10.1038/ncomms9087 (2015).

## Supplementary Material

Supplementary Information Supplementary Figures 1-13
