# Supplementary material for: Nek2 activation of Kif24 ensures cilium disassembly during the cell cycle
Source: Nat Commun. 2015 Aug 20;6:8087. doi: 10.1038/ncomms9087 (PMC4545512; doi:10.1038/ncomms9087)
Supplement: Supplementary Information — Supplementary Figures 1-13 [file ncomms9087-s1.pdf]

Supplementary Information

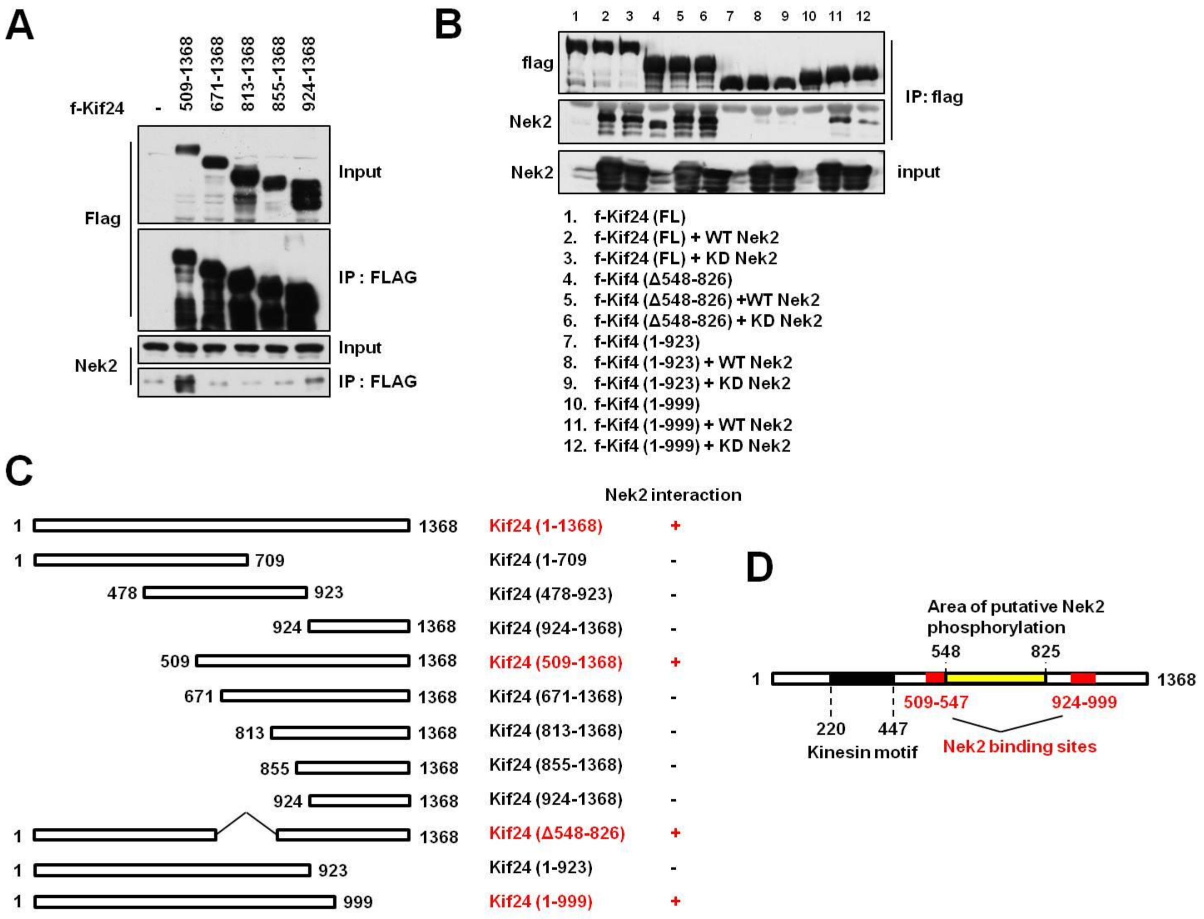

Supplementary Figure 1. Nek2 interacts with Kif24 through two regions encompassing residues 509-547 and 924-999. Lysates of HEK293 cells transfected with indicated Kif24 mutants (summarized in panel **c**) were immunoprecipitated with a Flag antibody and immuno-blotted with indicated antibodies to identify Nek2 interaction sites of Kif24 **(a-b)**. Schematic diagram of Kif24 highlighting the domains of interest **(d)**. Yellow region indicates Kif24 residues that are most highly phosphorylated by Nek2 **(d)**.

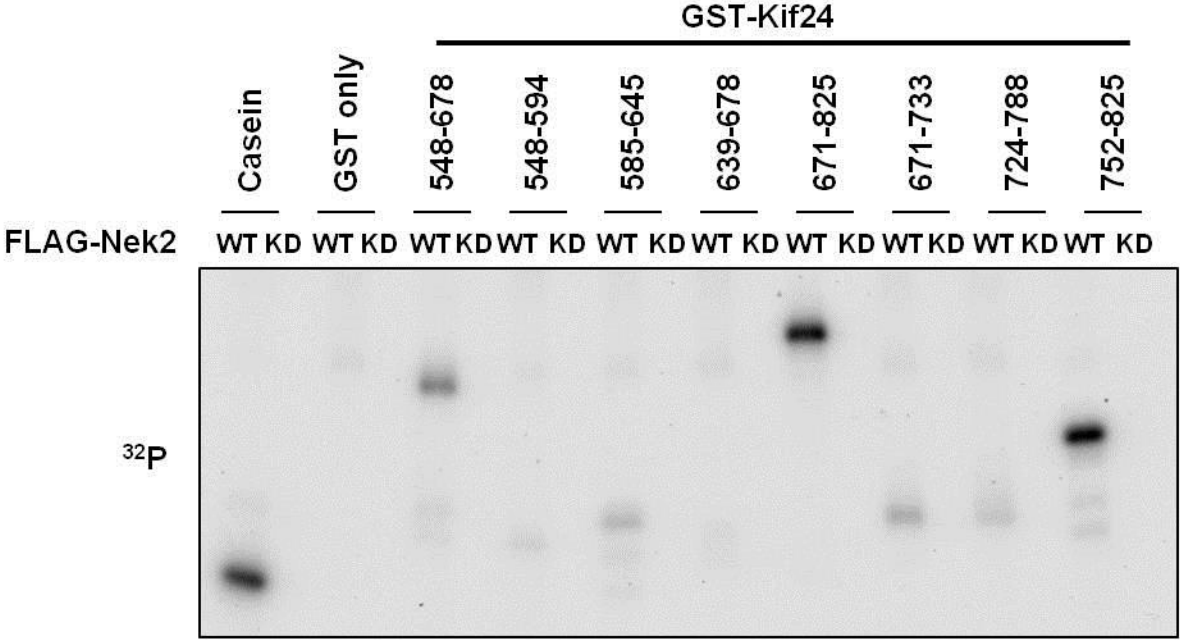

Supplementary Figure 2. Nek2 phosphorylates distinct sites within residues 548-825 of Kif24. *In vitro* kinase assays were performed with purified, recombinant wild-type or inactive Nek2 kinase and the indicated fragments of Kif24. Casein was used as postive control and GST only as negative control.

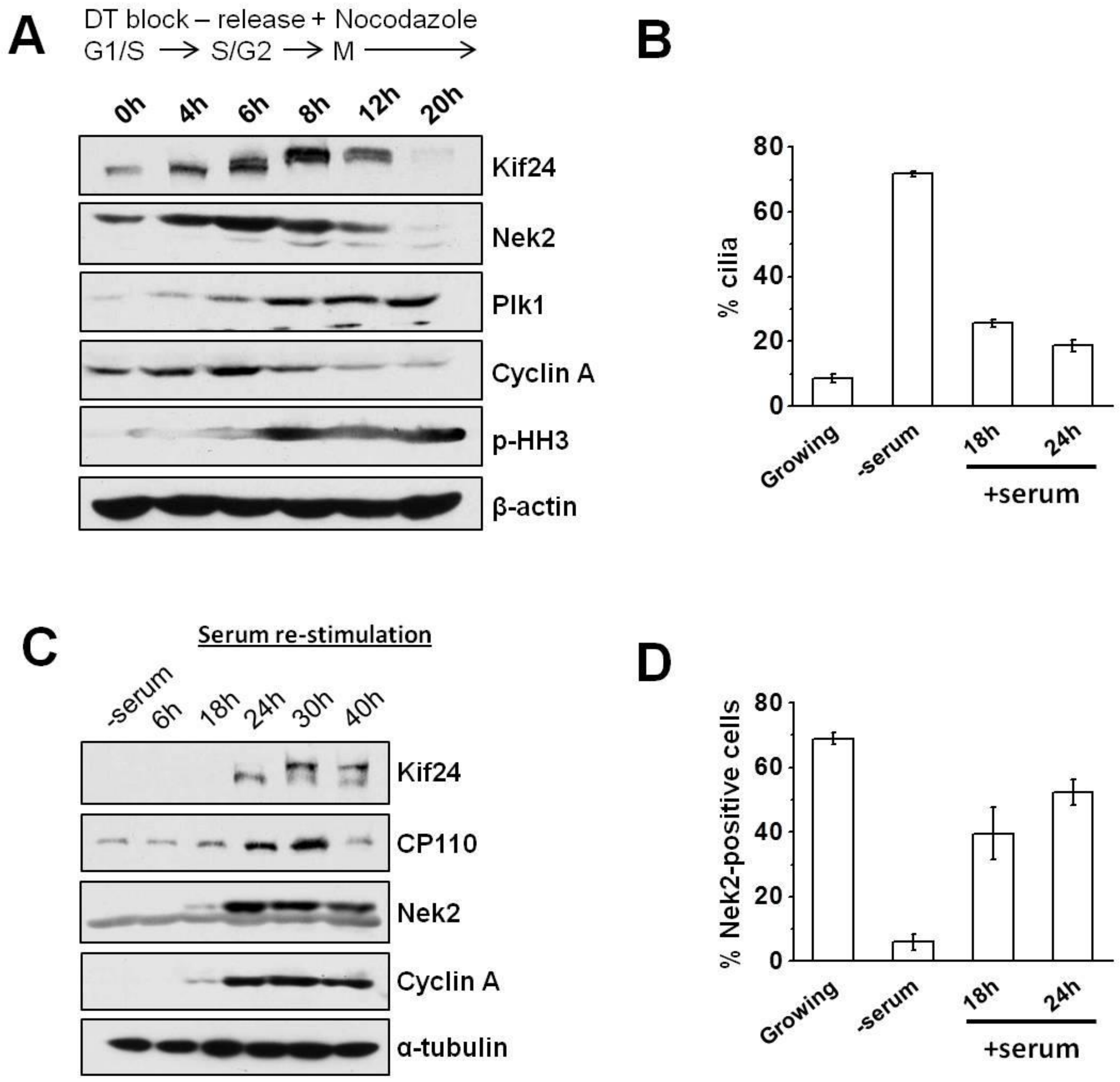

Supplementary Figure 3. Cell cycle dependent expression of Kif24 and Nek2 is incompatible with primary cilia formation. Kif24 is sequentially phosphorylated during the S/G2 transition in cells released from a double-thymidine (DT) block (**a**) and in cells re-stimulated from quiescence (**c**), concomitant with maximal expression of Nek2. Quantification of ciliation frequency (**b**) and Nek2 expression (**d**) in growing, serum starved (serum-), and serum re-stimulated populations. Data were obtained from n=3 biologically independent experiments. Error bars show SEM.

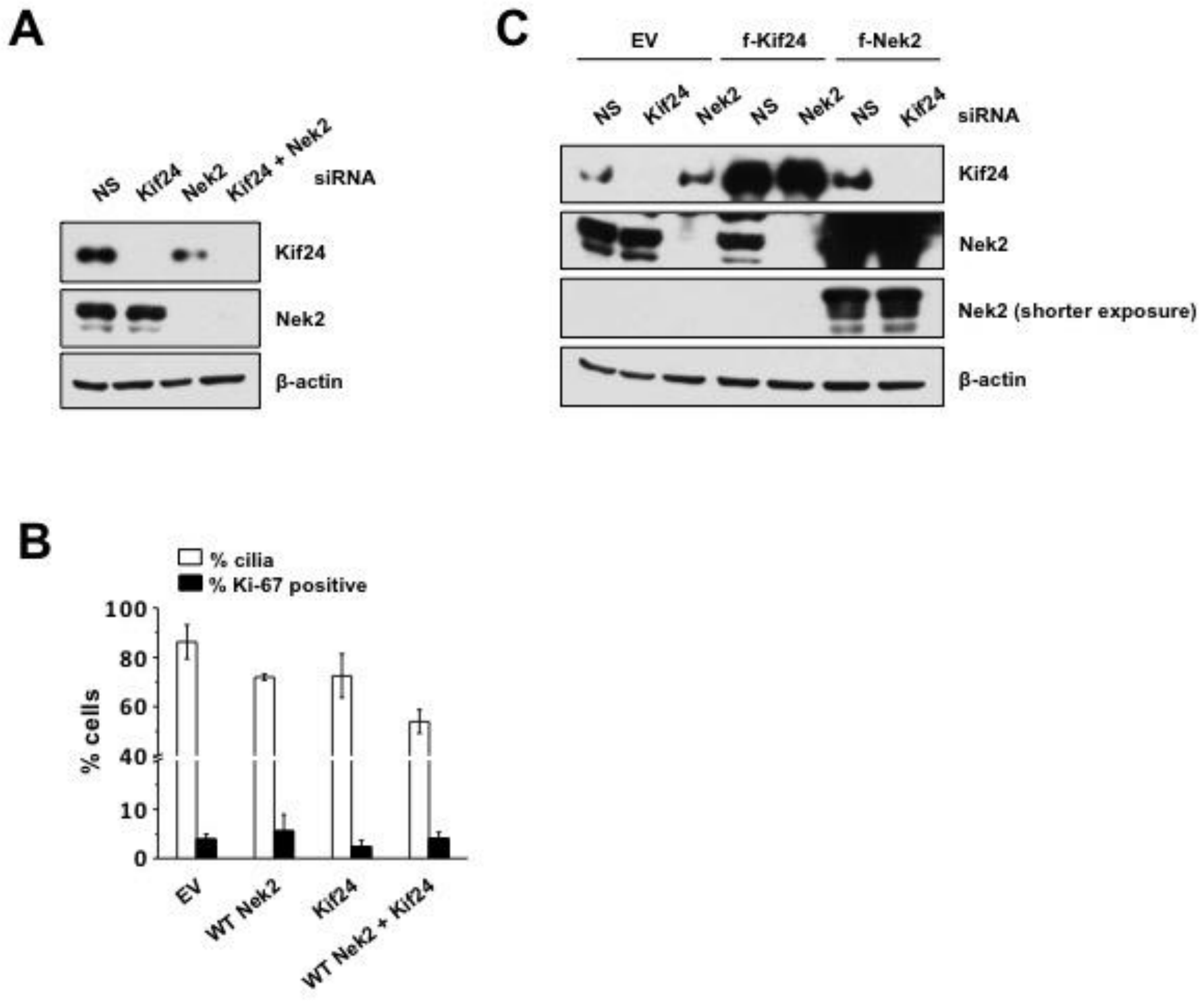

Supplementary Figure 4. Kif24 and/or Nek2 siRNA treatment mediates efficient knockdown. (a and c) Immuno-blot to determine knock-down efficiency for Figures 1A and C. **(b)** Ki-67 positivity (black bar) and frequency of ciliation (white bar) was assessed after expression of Kif24, Nek2, or both proteins at 48h of serum starvation. Data were obtained from n=2 biologically independent experiments. Error bars show SD.

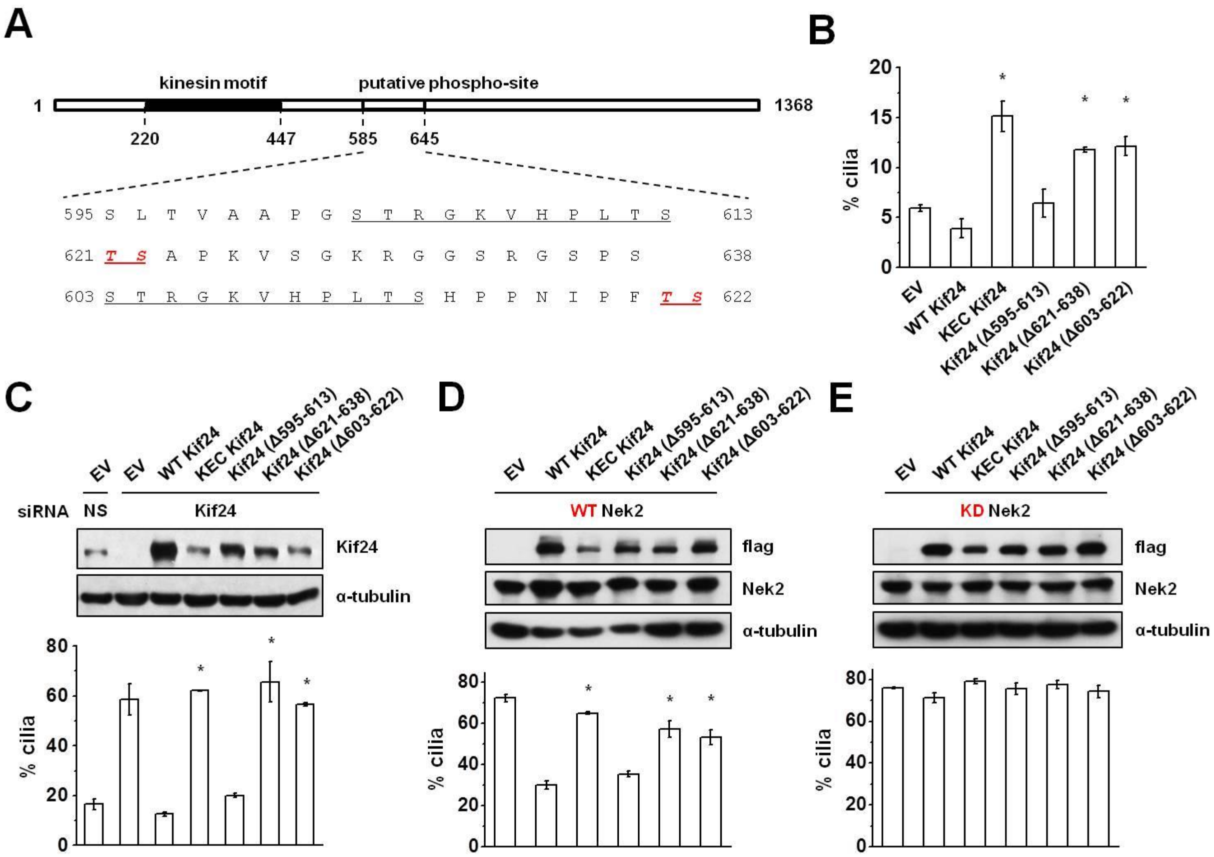

Supplementary Figure 5. Identification of critical Nek2 phosphorylation sites in Kif24 regulation. (**a**) Schematic showing Kif24 deletion mutants whose end-points are indicated. Underlined residues indicate overlapping areas between the internal deletion mutants of Kif24. Putative Nek2 phosphorylation sites are indicated in red. (**b-e**) Frequency of ciliation in RPE1 cells was measured after expression of deletion mutants depicted in panel (**a)**. Frequency of ciliation in RPE1 cells was examined after expression of indicated Flag-Kif24 deletion mutants combined with depletion of Kif24 in growing cells (**c**) or expression of WT (**d**) or kinase-inactive (KD) (**e**) Nek2 in serum-starved cells. Western blots of the resulting extracts were probed with antibodies against, Kif24, Flag, and Nek2. α-tubulin was used as loading control.

Data were obtained from n=3 biologically independent experiments. Error bars show SEM. *, $p<0.05$ (statistical significance tested against WT Kif24).

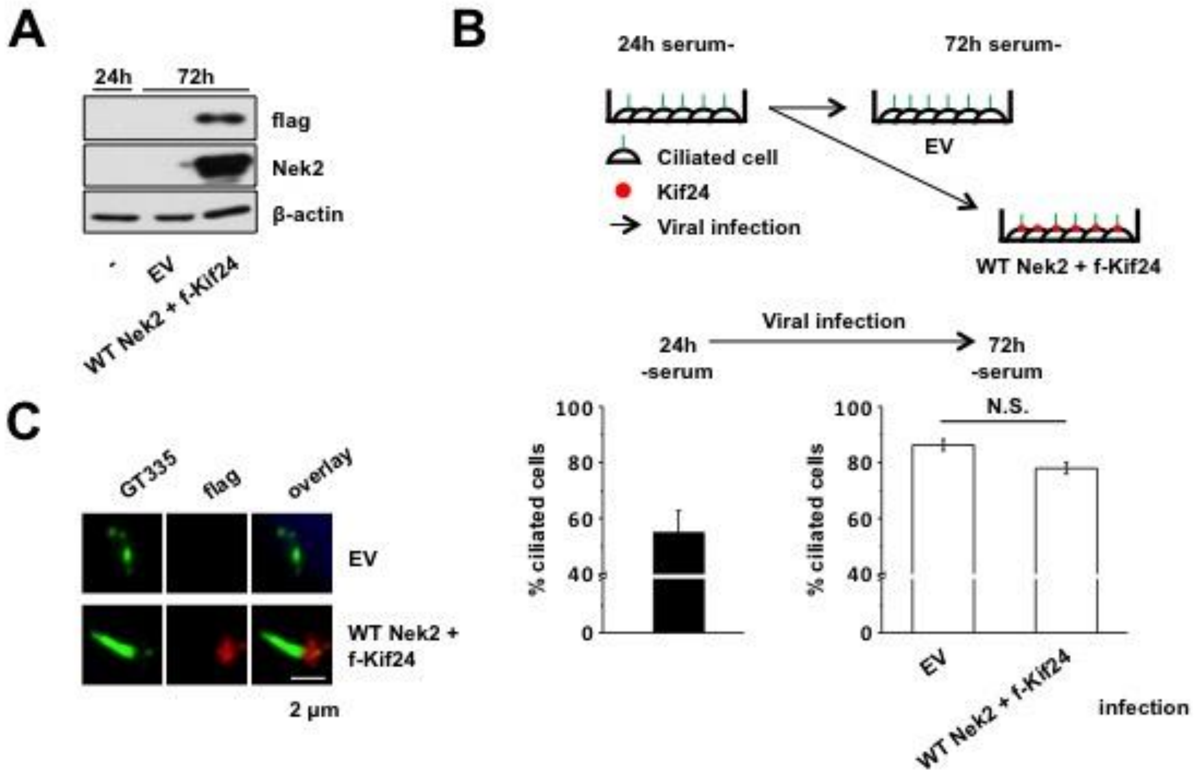

Supplementary Figure 6. Nek2-activated Kif24 does not disassemble fully formed ciliary axonemes. (a-c). Empty vector (EV, negative control) lentivirus or lentiviruses expressing flag-tagged WT Kif24 (f-Kif24) + WT Nek2 were used to infect RPE1 cells that were previously starved for 24h (serum-), as depicted in the schematic diagram (top panel **b**). Expression of the exogenous genes was determined, 48h post-infection in serum-free media by western blot **(a)** and immunofluorescence microscopy **(c)**. At 24h of serum deprivation (before infection), ~55% of RPE1 cells were ciliated, and ~86% and ~78% ciliation of EV or WT Nek2 + f-Kif24 expressing cells, respectively, were ciliated at 72h of serum deprivation **(b)**. Error bar indicates SD (n=2).

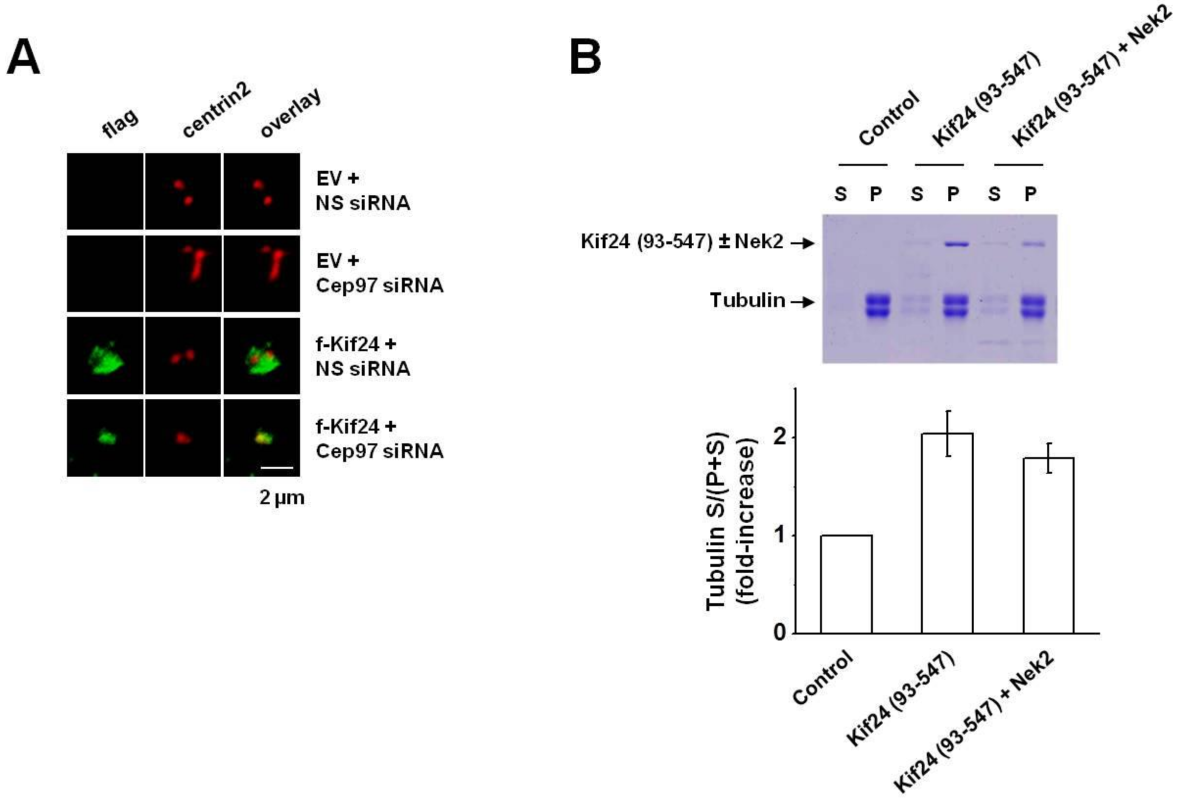

Supplementary Figure 7. Ectopic expression of Kif24 prevents abnormal centriolar elongation through its kinesin motif, which itself does not phosphorylated by Nek2. (**a**) Representative image of elongated centriole as determined by centrin-2 immuno-staining. Scale bar: 2 µm. (**b**) (*Top*) Coomassie gel showing the components of microtubule de-polymerization assay. (*Bottom*) Quantitation of results at top. A Kif24 fragment containing only the kinesin domain is impervious to stimulation by Nek2. Data were obtained from n=2 biologically independent experiments. Error bars show SD.

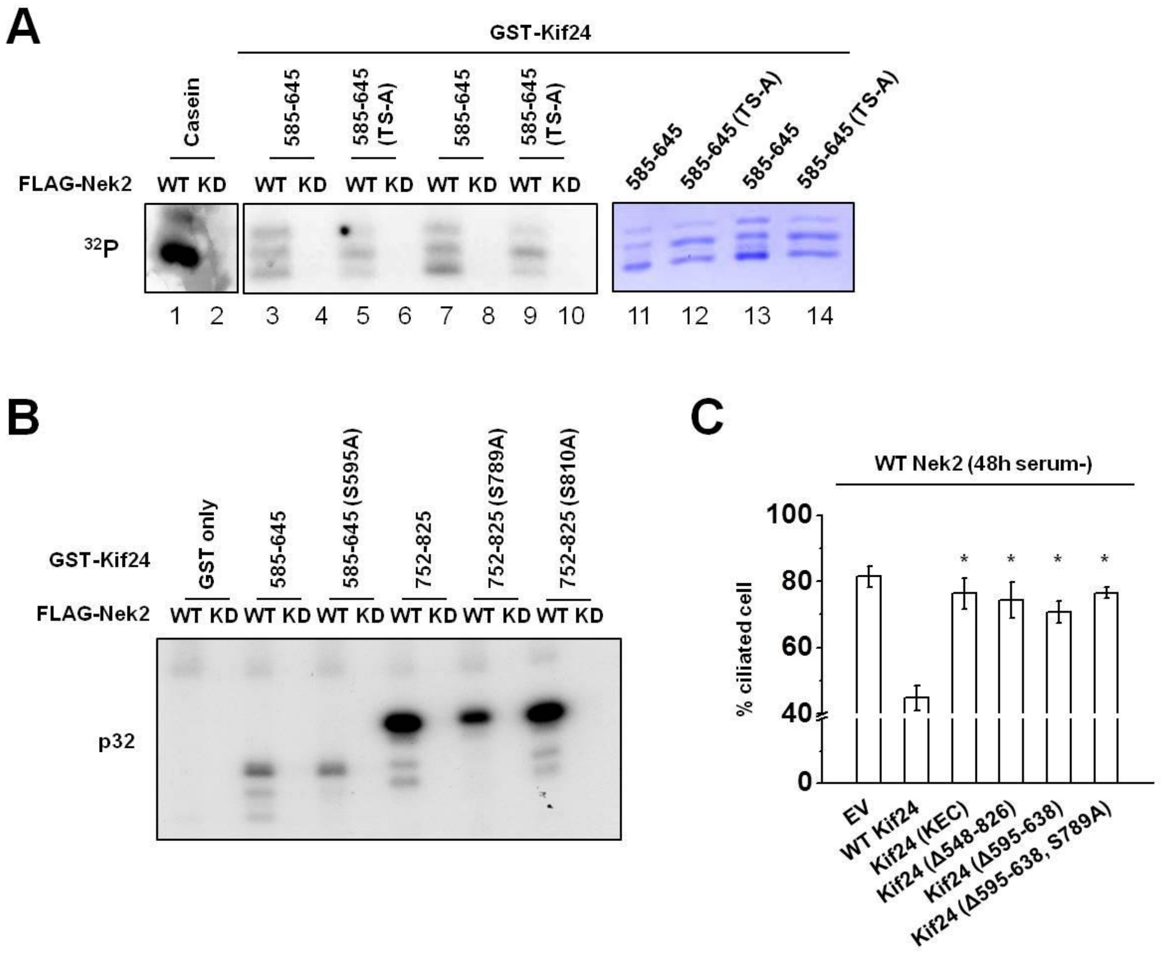

Supplementary Figure 8. **Kif24 residues T621 and S622, constitute important Nek2 regulatory sites. (a)** *In vitro* kinase assay to determine whether T621 and S622 are Nek2-dependent phosphorylation sites. Increasing amounts of substrate (shown on Coomassie gel, right) were used in the kinase assay (1x, lanes 3-6, 11, 12) and (1.5x, lanes 7-10, 13, 14)). Casein was used as positive control (lanes 1-2). (**b**) *In vitro* kinase assay to determine additional Nek2-dependent phosphorylation sites. (**c**) By comparing ciliation of 48h serum starved WT Nek2-expressing RPE1 cells, infected with EV (control), WT Kif24, Kif24 (KEC), Kif24 (Δ548-826), Kif24 (Δ595-638), and Kif24 (Δ595-638, S789A), we find that the frequency of ciliation between Kif24 (Δ595-638), and Kif24 (Δ595-638, S789A) are similar. This suggests that the

Kif24 residue S789 does not affect ciliation. Error bar indicates SD. *, p<0.05 (p-value was assigned in comparison to WT Kif24) (n=2).

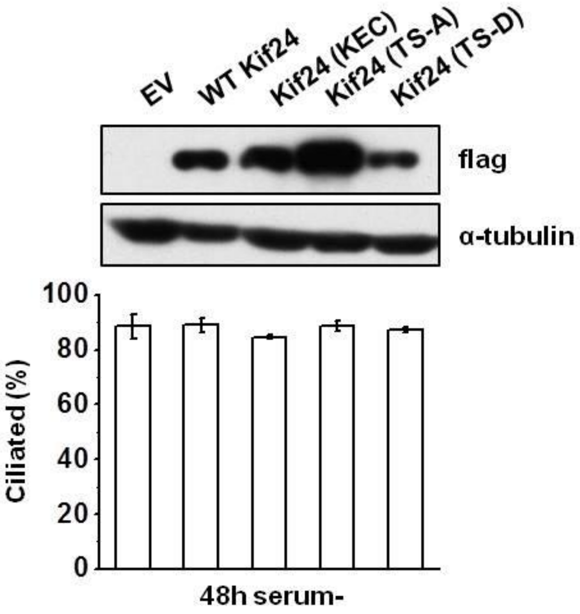

Supplementary Figure 9. Phospho-mimetic mutant of Kif24 does not have constitutive activity. RPE1 cells were infected with Lentivirus to express EV (control), WT Kif24, Kif24 (KEC), Kif24 (TS-A), and Kif24 (TS-D). One day post-infection, the cells were serum deprived for 48h and compared for their level of ciliation. Immunoblot shows ectopically expressed proteins as indicated using antibodies against Flag and α-tubulin as loading control. Error bar indicates SD. (n=2)

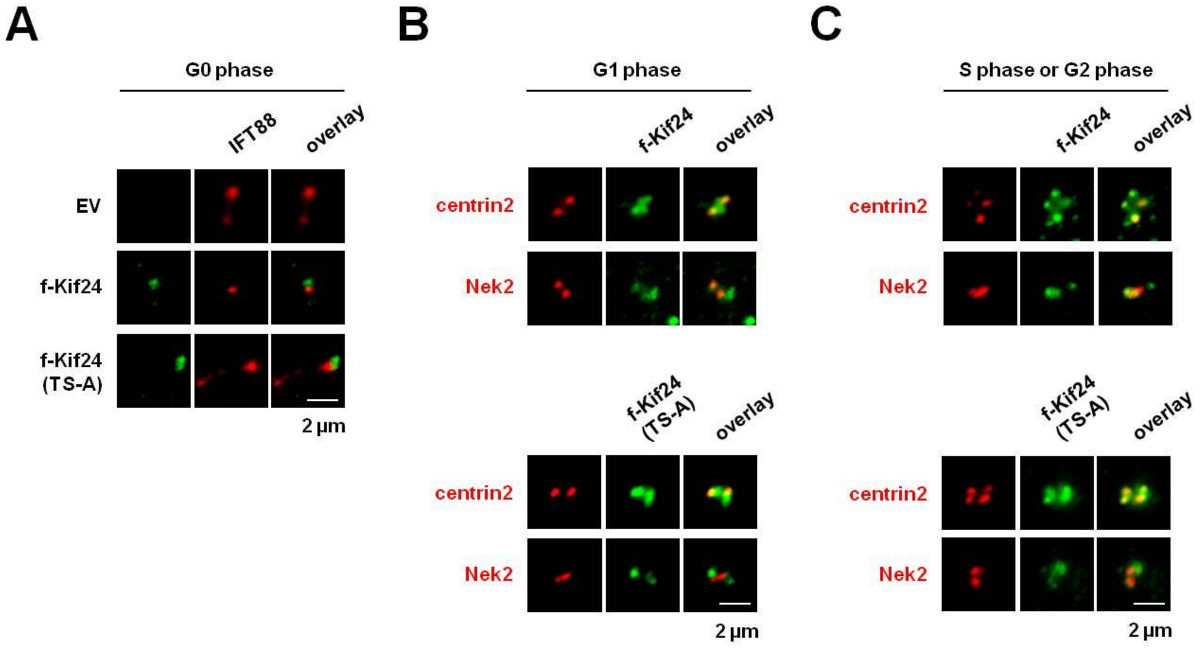

Supplementary Figure 10. Impaired ciliary function of Kif24 (TS-A) mutant is not due to mislocalization. (a-c) Localization pattern of ectopically expressed Kif24 or Kif24 (TS-A) was visualized using a ciliary marker (IFT88) after 48h serum starvation (G0) **(a),** and centrin-2 (distal centriolar marker) or Nek2 during G1 **(b)** and S or G2 **(c)** phase of the cell cycle. Scale bar: 2 µm.

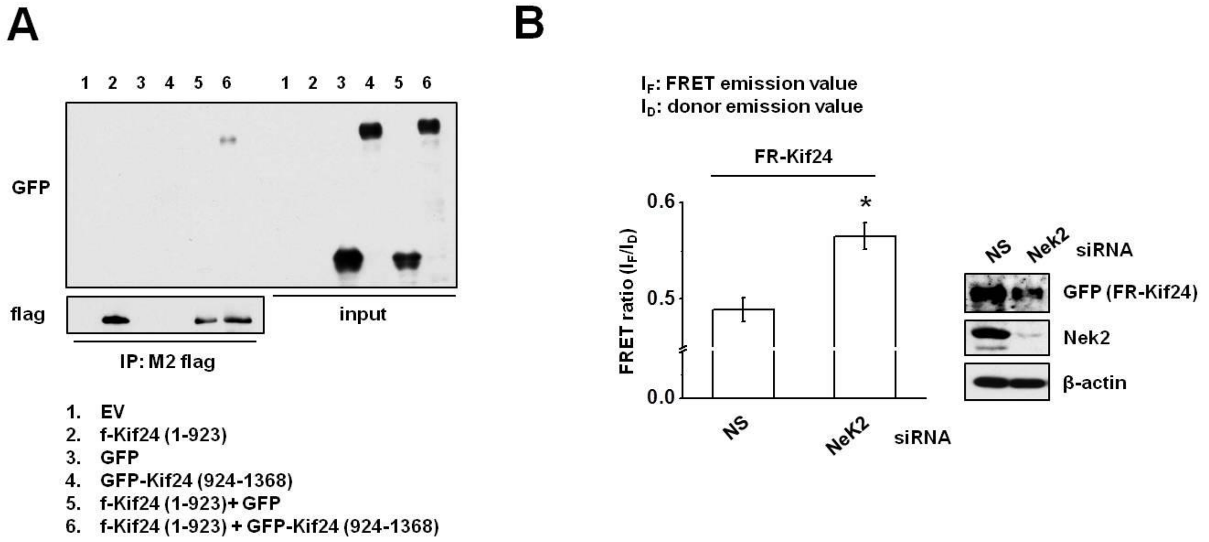

Supplementary Figure 11. The amino- and carboxy-terminal of Kif24 interact to form a 'closed' conformation, which is regulated by Nek2. (a) Extracts of HEK293 cells were immunoprecipitated and immuno-blotted with the indicated antibodies to demonstrate interaction between Kif24 (1-923) and Kif24 (924-1368). (**b**) Quantification of FRET emission ratio (I_F/I_D) of FR-Kif24 in cells depleted of Nek2. The immunoblot (*right*) shows efficiency of Nek2 siRNAs and expression of the FR-Kif24 probe. β-actin was used as loading control. Data were obtained from three biologically independent experiments. Error bars show SEM. *, $p<0.05$.

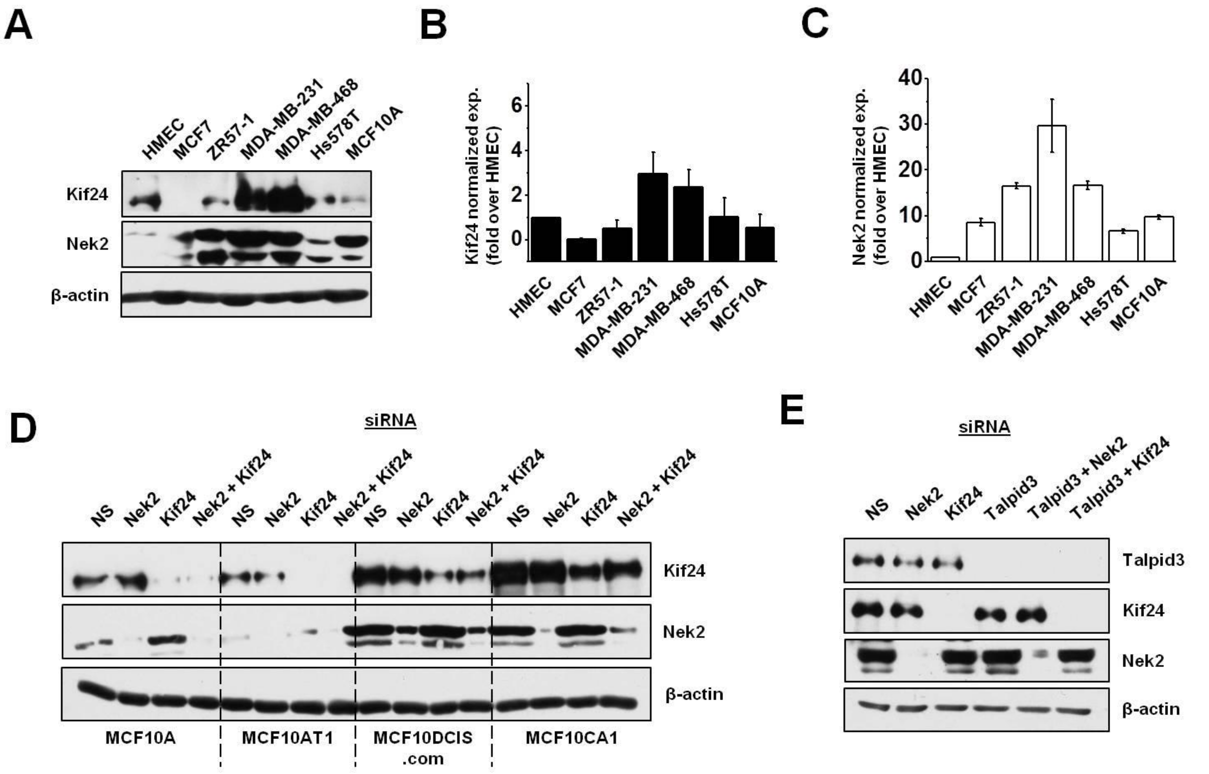

Supplementary Figure 12. Nek2 expression is upregulated in breast cancer cells (a) Expression of Kif24 and Nek2 was assessed in a panel of normal mammary (HMEC) and breast cancer cell lines. (**b and c**) Densitometric analysis of Kif24 **(b)** and Nek2 **(c)** expression, which was quantified using ImageJ software. Error bars indicate SD (n=2). (**d**) Kif24 and Nek2 expression was assessed by western blotting extracts of the MCF10 cell line series with and without depletion of either or both proteins, as indicated. **(e)** Immuno-blot to determine knockdown efficiency of Talpid3, Kif24 and Nek2 for Figure 7D and E.

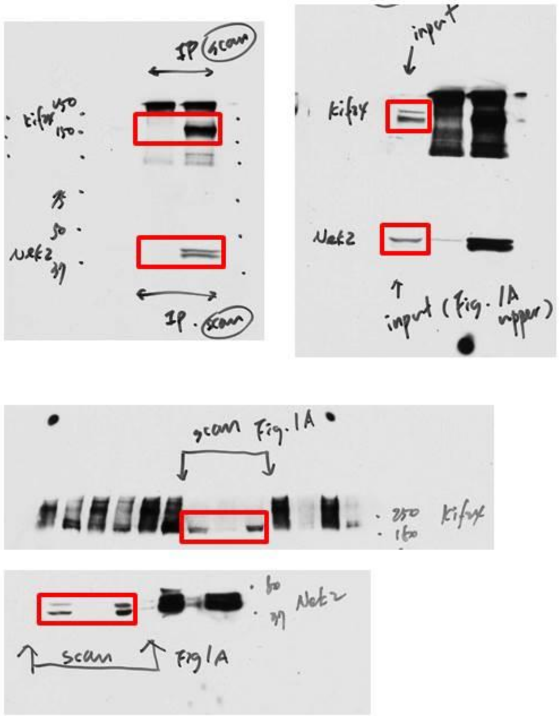

Fig. 1A

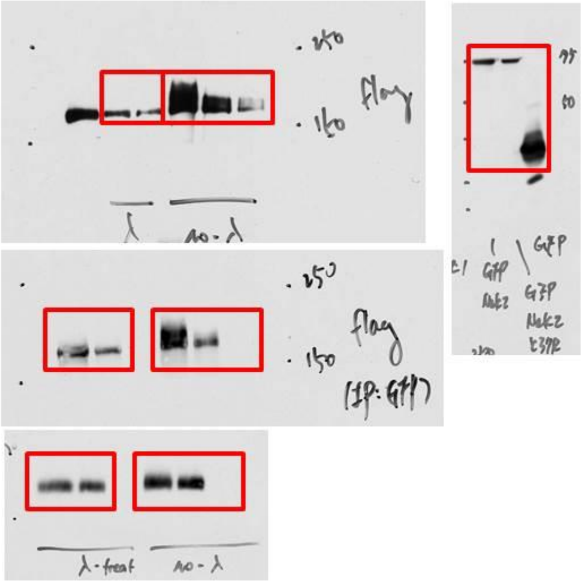

Fig. 1B

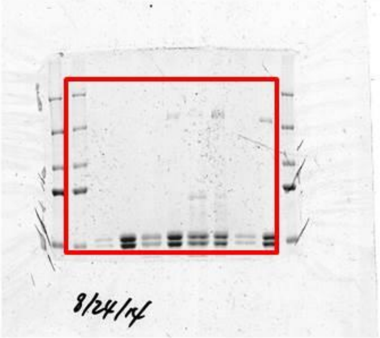

Fig. 3E

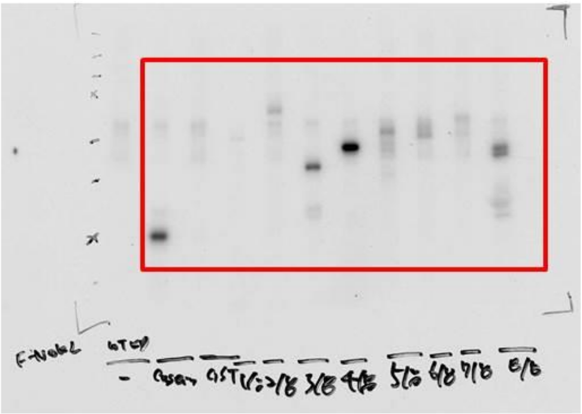

Fig. 1C

Supplementary Figure 13. Selected images of Western blots used in main figure.

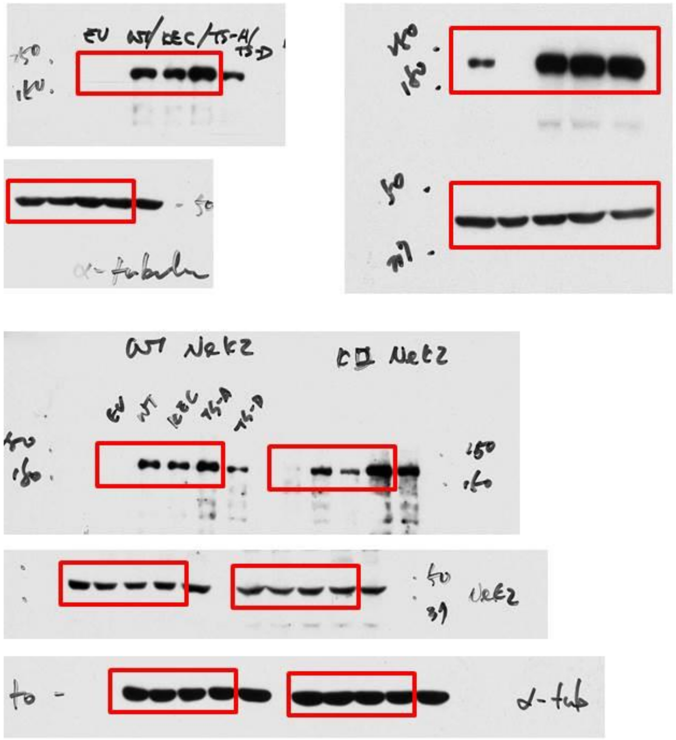

Fig. 4

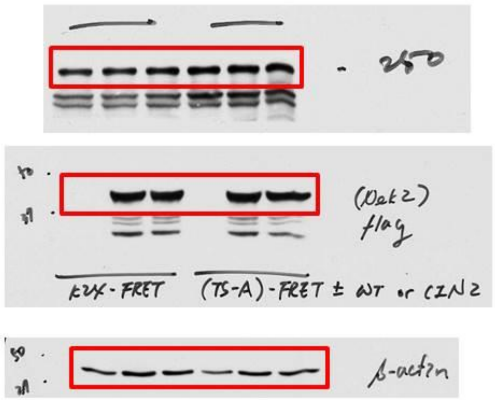

Fig. 5

Supplementary Figure 13. Selected images of Western blots used in main figure. (cont'd)
